# Numerical simulation of time-resolved 3D phase-contrast magnetic resonance imaging

**DOI:** 10.1371/journal.pone.0248816

**Published:** 2021-03-26

**Authors:** Thomas Puiseux, Anou Sewonu, Ramiro Moreno, Simon Mendez, Franck Nicoud

**Affiliations:** 1 IMAG, University Montpellier, CNRS, Montpellier, France; 2 Spin Up, Strasbourg, France; 3 I2MC, INSERM UMR 1297, Toulouse, France; 4 ALARA Expertise, Strasbourg, France; Texas A&M University System, UNITED STATES

## Abstract

A numerical approach is presented to efficiently simulate time-resolved 3D phase-contrast Magnetic resonance Imaging (or 4D Flow MRI) acquisitions under realistic flow conditions. The Navier-Stokes and Bloch equations are simultaneously solved with an Eulerian-Lagrangian formalism. A semi-analytic solution for the Bloch equations as well as a periodic particle seeding strategy are developed to reduce the computational cost. The velocity reconstruction pipeline is first validated by considering a Poiseuille flow configuration. The 4D Flow MRI simulation procedure is then applied to the flow within an *in vitro* flow phantom typical of the cardiovascular system. The simulated MR velocity images compare favorably to both the flow computed by solving the Navier-Stokes equations and experimental 4D Flow MRI measurements. A practical application is finally presented in which the MRI simulation framework is used to identify the origins of the MRI measurement errors.

## 1 Introduction

It is now well-established that hemodynamics is associated with the onset and evolution of several cardiovascular disorders such as aneurysms, stenoses, or blood clot formation [1–3]. Over the recent years, there has been increasing interest in using time-resolved 3D phase contrast Magnetic Resonance Imaging (or 4D Flow MRI) [4] for detection and follow-up of numerous vascular diseases as well as for research purposes. In addition to providing comprehensive velocity data and vascular motion in a single exam, 4D Flow MRI also offers the possibility to retrospectively evaluate numerous biomarkers derived from the velocity field, such as the relative pressure field [5], the wall shear stress [6], or the pulse wave velocity [7]. However, several acquisition parameters (e.g.: spatio-temporal resolution, encoding velocity, imaging artifacts) may limit the expected accuracy of the measurements and potentially lead to erroneous diagnosis [8, 9]. Moreover, the intrinsic complexities of the multi-modal MRI acquisition process make it delicate to localize the sources of the measurement errors. The signal processing steps required to reconstruct an MR image as well as the large variety of user-dependent acquisition parameters are as many potential sources of errors that could alter the measurements, and possibly lead to misdiagnosis. The numerical simulation of the MRI acquisition process could be an efficient way to decompose the acquisition process and to understand the mechanisms leading to measurement errors. It has already proven useful to describe and correct some sources of imaging artifacts [10], as well as to optimize sequences [11] for anatomical MRI. The recent MR fingerprinting technique [12] is also a good illustration of a possible use of MRI simulation to generate a dictionary containing predicted MR signals for a representative variety of tissue in order to improve the quantification of material properties. In that respect, the numerical simulation of 4D Flow MRI could provide a better understanding of the flow errors and help optimizing the sequences.

The main core of the acquisition process is based on the phenomenon of Nuclear Magnetic Resonance (NMR) which is described at the macroscopic scale by the Bloch equations [13]. These equations describe the macroscopic motion of the nuclear magnetization arising when a sample of nuclear spins (isochromat) experiences an external magnetic field. They read:

$$\begin{array}{c} \frac{dM(t)}{dt}=\gamma M\left(t\right)\times B\left(t\right)+\frac{M_{0}-M_{z}\left(t\right)}{T_{1}}{\hat{e}}_{z}-\frac{M_{x}\left(t\right)}{T_{2}}{\hat{e}}_{x}-\frac{M_{y}\left(t\right)}{T_{2}}{\hat{e}}_{y}, \end{array}$$ (1)

where *γ* is the gyromagnetic ratio, **B** is the external magnetic field experienced by the isochromat, **M** = (*M*_*x*_, *M*_*y*_, *M*_*z*_) is the nuclear magnetization vector, *T*_1_ and *T*_2_ are the relaxation times of the magnetization and $M_{0}{\hat{e}}_{z}$ is the steady-state magnetization. An isochromat refers to a sample of spins large enough to be described by the macroscopic Bloch equations, with similar position (*x*, *y*, *z*) at time *t*, magnetic properties (*T*_1_,*T*_2_, *M*_0_) and precession frequency (see Sec. 2). Although *T*_1_, *T*_2_, *M*_0_ characterize the macroscopic nature of a tissue, the gyromagnetic ratio is an atomic property. Note also that the hydrogen atom (for which *γ* = 267.5 × 10^6^ rad/s/T) is most often exploited in MRI as it is the most abundant atomic element in the human body.

Although many simulation frameworks have already been developed for static tissues imaging [14–17], flow MRI modeling is still a challenging issue. This is mainly due to the necessity to account for the dynamics of the spins, which results in a considerable increase of the computational load. In its classical formulation (Eq 1), the Bloch equations are defined for each isochromat, for which they are ordinary differential equations expressed in a Lagrangian formalism. Nevertheless, when simulations with moving spins are targeted, the input velocity field required to update their position is usually predicted by Computational Fluid Dynamics (CFD) on a fixed Eulerian numerical mesh.

A classical approach often adopted in the literature consists in solving the Eulerian formulation of the Bloch equations [18–20]. In this case, the CFD velocity (**u**) is used to transport the magnetization vector and a convection term is explicitly added to the time rate of change of the magnetization vector (**M**), which becomes:

$$\begin{array}{c} \frac{dM}{dt}\left(r,t\right)=\frac{\partial M(t)}{\partial t}+\left(u\left(r,t\right)\cdot \nabla \right)M\left(t\right). \end{array}$$ (2)

This approach has a relatively low computational cost since both the flow and Bloch equations can be solved on the same fixed mesh with no velocity interpolation needed. Nevertheless, the Eulerian Bloch equations are partial derivative equations that do not admit a generally valid analytical solution. Moreover, the Eulerian formalism encompasses some modeling assumptions as the necessity to prescribe boundary conditions for the magnetization vector. Note also that transformations of the mesh are often used to correct the results for the spatial misregistration effects [18, 19], although some alternatives use local magnetization transformations to account for the flow-related effects [21]. Finally, the Eulerian approach is less adapted to complex flow configurations, where the time scale of the velocity variations may be small as compared to the time scale of the MR sequence [19].

An alternative approach consists in modeling the spin isochromats with Lagrangian particles, using the CFD velocity to update each particle position. The Bloch equations can then be solved independently for each particle, with no spin-spin interaction [22–24]. Therefore, the computational load can easily be partitioned on multiple cores to accelerate the calculations. Nevertheless, a sufficient number of particles is required to accurately approximate the MR signal. As discussed by Shkarin & Spencer [25], at least 3 isochromats/direction/voxel are necessary to reduce the MR signal error to 1.5%: this may require high computational resources depending on the image spatial resolution (450 million particles for a 256 × 256 × 256 image). Since homogeneous particle repartition within the domain is also required to avoid zones with spurious MR signals [24], the existing studies are most often limited to simulations in simple geometries [18, 20, 26]. Moreover, in the usual procedure [15, 21–24, 26, 27], a prior CFD simulation is performed to store all the particle positions during the entire simulation. This approach can be suited to steady flows but seems irrelevant for pulsatile flows simulations in which very long physical times, and thus many particle positions, generally need to be simulated. For example, a particle tracking along a 4D Flow MRI simulation of physical scan duration *T*_*acq*_ = 6 min, discretized with a constant time step Δ*t*_*CFD*_ = 10^−3^ s, and with 3 particles/direction/voxel injected would require to store about 60 TB of memory for an acquisition matrix of size (160 × 160 × 20). The huge file size, the repeated accesses to this file, and the temporal interpolations required to update the particles position at different instants during the MRI simulation, would lead to prohibitive computational costs. To address this problem, we propose a method to perform MRI/CFD simulations “on the fly”, i.e. advancing the particle positions and computing the resulting NMR signal during the calculation, without storing the particle trajectory history.

Well-resolved computations are now achievable at reasonable costs because of computational power gains due to the recent improvements of hardware and software capabilities. To that extent, Lagrangian computations are now feasible and adapted to simulate complex flow MRI measurements [19]. However, to the best of the authors’ knowledge and as summarized in the literature review presented in Table 1, no simulation framework of 4D Flow MRI (or time-resolved 3D PC-MRI) sequences has ever been proposed yet.

**Table 1:** Review of the published works in flow MRI simulations.

Publication	Configuration	Formulation	Sequence
Steinman et al., 1997 [27]	steady 3D idealized bifurcation	Lagrangian	2D/3D GE VE
Jou et Saloner, 1998 [18]	pulsatile 2D carotid bifurcation	Eulerian	2D GE VC/VU
Lorthois et al., 2005 [19]	steady 2D carotid bifurcation	Eulerian	2D GE VC/VU
Marshall, 2010 [23]	steady 3D carotid bifurcation	Lagrangian	3D GE VE
Petersson et al., 2010 [22]	steady 3D stenosis	Lagrangian	3D GE VE
Jurczuk et al., 2014 [28]	steady 3D vascular network	Eulerian	2D/3D GE
Xanthis et al., 2014 [29]	steady 3D cylinder	Lagrangian	2D GE VE
Klepaczko et al., 2014 [26]	steady 3D stenosed/U-bend tubes	Lagrangian	3D GE VC
Fortin et al., 2018 [24]	steady 3D cerebral artery	Lagrangian	3D GE VE

GE: gradient echo; VC: velocity compensated; VU: velocity uncompensated; VE: velocity encoded. Detailed explanations on the sequence terminologies are given in Sec. 2.4 and 2.5.

The objective of this work is to present a workflow able to simulate time-resolved 3D PC-MRI acquisitions of flow fields of arbitrary complexity. Pulsatile transitional flows in complex geometries, as encountered in the cardiovascular system, are specifically targeted. To this aim, an original framework is proposed where the Bloch equations are advanced on Lagrangian particles behaving like tracers in a flow field solved simultaneously. Hence, the Bloch equations are solved “on the fly” so that the particles trajectories do not need to be stored, as usually done [23, 24]. The resulting simulated MR signal is collected and synthetic MR images are reconstructed. As the simulated MR images are calculated from a reference flow field, the comparison between the flow field predicted by CFD and that reconstructed by the simulated MRI (SMRI) process allows the identification of the sources of errors due to the imaging technique. In particular, it is virtually possible to add assumptions and sources of errors to quantify their effect on the results.

To illustrate the potential of the method and compare with real MRI measurements, a well-controlled experiment delivering a pulsatile blood-mimicking fluid flow within a rigid phantom typical of the cardiovascular system was designed and several PC-MRI experiments were carried out [30]. An image-based CFD analysis was performed, prescribing as inlet velocity profile the PC-MRI measurements performed. The Bloch equations were also solved in this configuration, in order to compare standalone CFD and SMRI results with MRI experimental measurement. As we have full control of the geometry of the non deformable flow domain and fluid rheology, classical sources of uncertainties met *in vivo* such as segmentation errors, wall motion and blood properties are suppressed, which potentially enables to identify the sources of errors coming from the MRI process itself.

Some basic concepts of Magnetic Resonance Imaging intended to the non-expert readers are first introduced in Sec. 2. The numerical procedure for simulating the Bloch equations as well as the coupling strategy with CFD are then presented in Sec. 3. The verification and validation of the developed numerical pipeline are detailed in Sec. 4. The Bloch equations solver is first validated by reproducing the numerical test case published in Yuan et al. [31]. Then, the full velocity reconstruction pipeline is verified through the MRI simulation of a Poiseuille flow configuration. The CFD coupling is finally validated through the full 4D Flow simulation of the flow phantom experiment described above [30]. The reconstructed images are compared with both the input CFD velocity maps and experimental 4D Flow MRI measurements. The influence of the spatial resolution and particle density on the reconstructed velocity field is also investigated.

## 2 Basic concepts in MRI

The next section presents MRI concepts that are essential for the understanding of the proposed method by non-expert readers.

### 2.1 Nuclear Magnetic Resonance (NMR)

**Fig 1:**
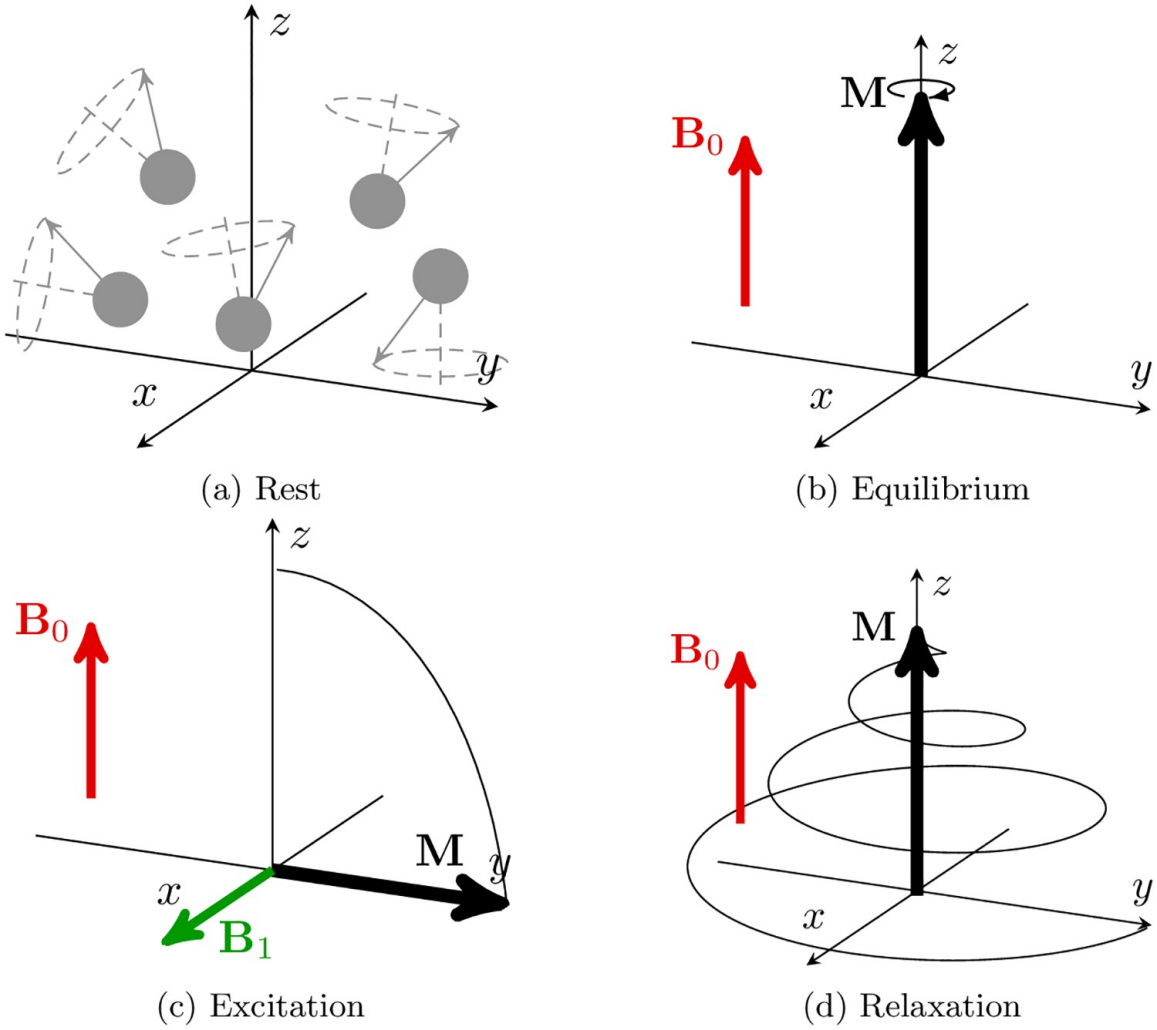
Illustration of the NMR experiment. (a) When no magnetic field is applied, the spins are randomly oriented. When a **B**_0_ field is applied along the z-axis, all the spins precess around the z-axis and (b) an equilibrium magnetization arises, oriented along the same axis. The equilibrium magnetization is shifted towards the transverse xy-plane by the effects of an RF-pulse (*B*_1_) applied at resonance frequency (c). When the RF-excitation is released, the magnetization relaxes towards its equilibrium value (d) with a precession frequency that depends on the magnetic properties of the isochromat considered. Note that the magnetization shift due to the RF-pulse is around two or three orders of magnitude faster than the relaxation process.

The NMR experiment is illustrated in Fig 1. Without any external magnetic field, the nuclear spins (i.e the total angular momentum of an atomic nucleus) are randomly oriented (Fig 1a). However, if a uniform static magnetic field (**B**_0_) is applied to a sample of spins, the spins start precessing at the Larmor frequency *ω*_0_ = *γB*_0_ around the **B**_0_ axis and an equilibrium magnetization vector **M** = **M**_0_ aligned with **B**_0_ arises from the sum of their magnetic moments (Fig 1b). The NMR experiment mainly consists of an excitation of the sample of spins from their equilibrium state associated with *B*_0_, followed by a relaxation in which a tissue-specific magnetic signature is collected. The excitation consists in applying a radiofrequency (RF) pulse at Larmor frequency to disturb the net magnetization vector from its equilibrium state and shift it towards the transverse plane (Fig 1c). At the end of the RF excitation, the spins sample, or isochromat, evolves towards its equilibrium state (Fig 1d) and the net magnetization resulting from the relaxation induces a temporal variation of the magnetic flux (according to Faraday’s law of induction) measured through a receiver coil during a readout event.

### 2.2 Signal reception

The signal measured by a receiver coil during the relaxation of the isochromat is proportional to the spin density of the excited sample. Formally, the complex signal can be written as:

$$\begin{array}{c} S\left(t\right)=\int_{\Omega }M_{xy}\left(r,t\right)C_{xy}\left(r\right)d\Omega , \end{array}$$ (3)

where the transverse magnetization (*M*_*xy*_ = *M*_*x*_ + *iM*_*y*_) is integrated over the whole sample Ω and *C*_*xy*_ is the receiver coil sensitivity profile; in this work, the coils are supposed perfect (*C*_*xy*_ = 1) over the entire domain.

### 2.3 Signal localization

In practice, the sample is made up of different tissues (e.g.: fat, liver, blood) with specific proton densities and relaxation properties (*T*_1_ and *T*_2_, see Eq 1). The overall received MR signal is therefore a sum of the net magnetization signals emitted by each tissue. To localize the spatial distribution of each tissue within the sample, an additional spatially varying magnetic field (gradient field) is added to the static field **B**_0_ thus linearly modifying the precession frequency of the spins in space. Subsequently, each isochromat contributes with its own frequency and phase to the signal recorded by the receiver coil. Three-directional magnetic gradients fields **G** = (*G*_*x*_, *G*_*y*_, *G*_*z*_) are applied in 3D imaging to encode the isochromats along each direction.

### 2.4 MR sequence

The temporal arrangement of the external magnetic field in the Bloch equations (see Eq 1) is referred to as the MRI sequence and mainly consists of repetitions of RF-excitations, phase and frequency encoding gradients and signal readouts. A repetition refers to this elementary series of events in the external magnetic field. To each instant *t* during the signal readout corresponds a particular phase and frequency encoding of the signal. Therefore, a unique wave number **k**(*t*) = (*k*_*x*_(*t*), *k*_*y*_(*t*), *k*_*z*_(*t*)) can be defined as:

$$\begin{array}{c} k\left(t\right)=\frac{\gamma }{2\pi }\int_{0}^{t}G\left(t\right)dt. \end{array}$$ (4)

where the time origin *t* = 0 is taken as the end of the first RF pulse. The space containing **k**(*t*) is referred to as the k-space and corresponds to the Fourier conjugate of the standard spatial domain Ω. Using this k-space formalism, an MRI sequence can be defined as the temporal arrangement of magnetic fields necessary to cover a specific trajectory in the discretized k-space. In the classical Cartesian k-space filling strategy, a phase-encoding gradient is first applied along *k*_*y*_ direction to encode the spins phase. A frequency-encoding gradient is then applied along *k*_*x*_ direction during the readout to modify the spin frequencies as the time increases. In other words, one k-space line in the *k*_*x*_ direction is filled during each signal readout, which itself consists of several readout samples. For 3D imaging, an additional phase-encoding gradient is applied along the *k*_*z*_ direction.

**Fig 2:**
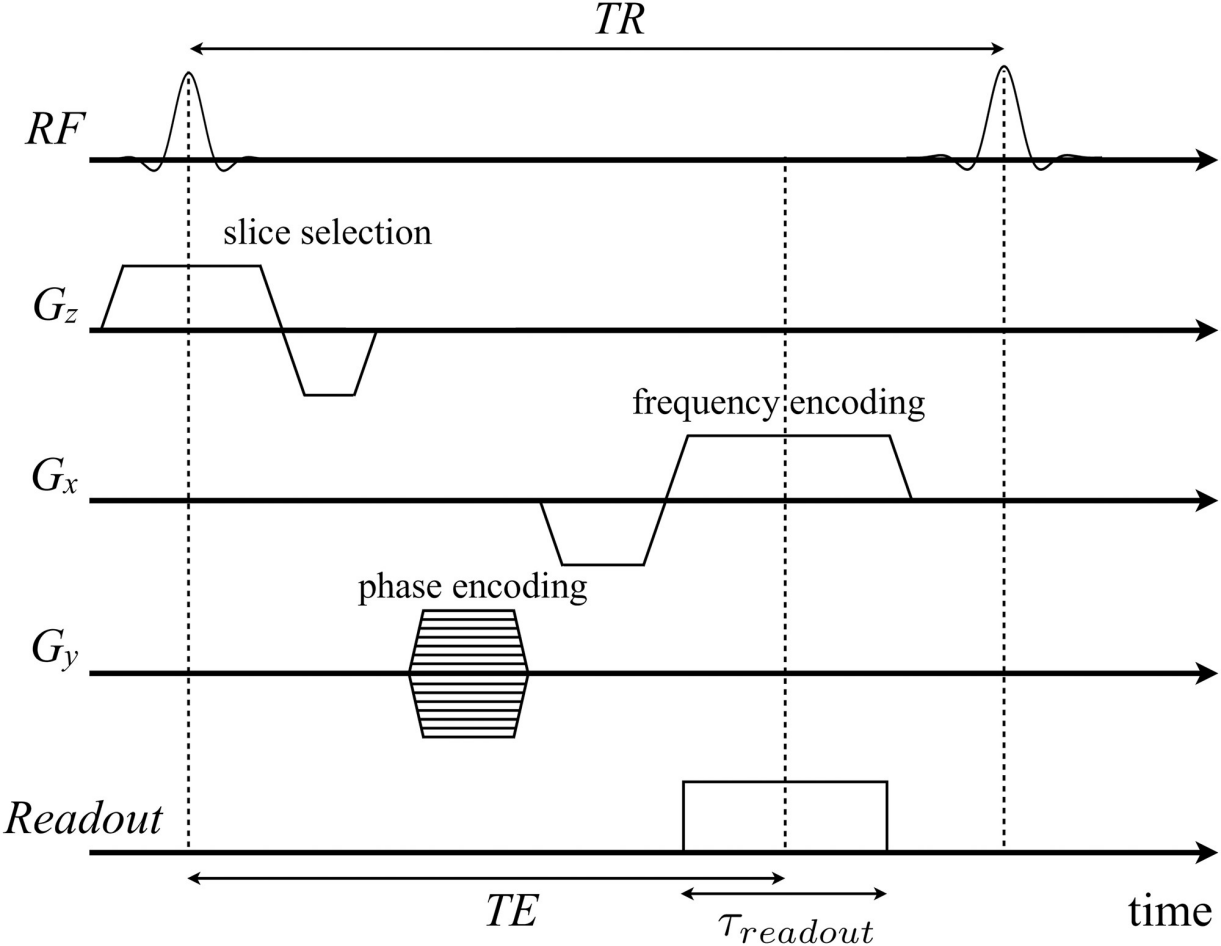
Diagram of a typical gradient echo pulse sequence with frequency and phase encoding gradients along *x*-axis and *y*-axis, and slice selection gradient along *z*-axis. Each k-space line is filled during the readout event where the signal is measured by the receiver coil. This pulse sequence is repeated changing the phase encoding gradient amplitudes (*G*_*y*_) after each repetition time *TR* to incrementally fill the k-space lines, as represented by the several amplitudes. For full Cartesian k-space sampling, the time between the RF-excitation and half the readout corresponds to the echo time and is denoted TE. It is a characteristic time of the evolution of the isochromats between the excitation by the RF and the signal measurement.

In practice, several groups of pulse sequences can be distinguished (spin echo, gradient echo, inversion recovery, …) to highlight specific anatomic or functional parameters; gradient echo pulse sequences are generally adapted to flow imaging. The chronogram of a typical gradient echo sequence is illustrated in Fig 2.

Formally, an MRI sequence is defined by the characteristics of the external magnetic field **B**(**r**, *t*) (see Eq 1). However, the Bloch equations are generally expressed in the frame of reference that rotates clockwise at Larmor frequency *ω* = *ω*_0_ = *γB*_0_, around the *z*-axis by convention. This cancels the static magnetic field contribution such that the magnetic field is referred to as the effective magnetic field $B_{\text{eff}}=B-\frac{\omega_{0}}{\gamma }{\hat{e}}_{z}$. The effective magnetic field at position **r** and time *t* in this frame of reference reads:

$$\begin{array}{c} B_{\text{eff}}\left(r,t\right)=(\begin{array}{c} B_{x}\\ B_{y}\\ B_{z} \end{array})=(\begin{array}{c} B_{1}\left(t\right)\cos\left(\omega_{1}t\right)\\ -B_{1}\left(t\right)\sin\left(\omega_{1}t\right)\\ r\cdot G\left(t\right)+\Delta B_{z}\left(r,t\right) \end{array}), \end{array}$$ (5)

where **B**_1_ is the RF field that rotates around the *z*-axis at frequency *ω*_1_ with respect to the frame of reference. Note that the RF pulse shape is often described by a filtered cardinal sine (SINC) function, as its Fourier transform corresponds to a rectangular profile. SINC RF pulses equally excite the spins within a given bandwidth while leaving the surrounding spins precessing at a frequency out of the given bandwidth unaffected. **r**⋅**G** in Eq 5 corresponds to the magnetic field induced by the gradient coils and Δ*B*_*z*_(**r**, *t*^*n*^) represents the deviations of the magnetic field due to off-resonance effects. The off-resonance effects mainly comprise non-linear gradients (non-constant **G**(*t*)), concomitant fields, eddy currents, chemical shift, $T_{2}^{*}$ dephasing and magnetic susceptibility. In the initial development phase, these effects are neglected, thus it is assumed that Δ*B*_*z*_ = 0 hereafter.

### 2.5 Phase-contrast MRI

Phase-contrast MRI exploits the relationship that exists between the phase shift of moving spins and their velocities. The phase shift encompassed by an isochromat between the RF-excitation (at *t* = 0) and a given time *t* can be written in the rotating frame of reference as:

$$\begin{array}{c} \phi \left(r,t\right)=\int_{0}^{t}\gamma B_{z}\left(r,t\right)dt=\gamma \int_{0}^{t}r\left(t\right)\cdot G\left(t\right)dt. \end{array}$$ (6)

**Fig 3:**
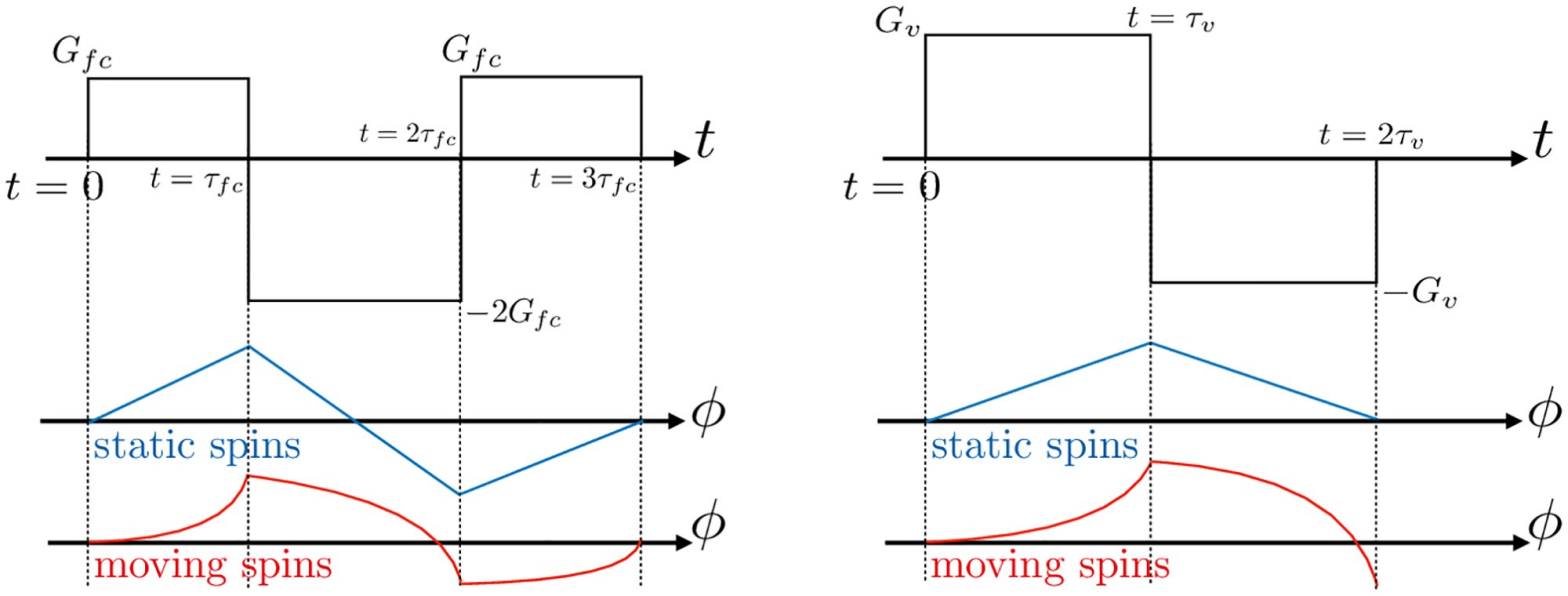
Illustration of the effects of applying either flow compensating (left) or bipolar encoding velocity gradients (right) on the phase of the magnetization vector. The static spins (blue) result in a zero accumulated phase at the end of the application, while the moving spins (red) result in a non-zero phase.

In particular, it can be written for *t* = *TE*, the echo time, which is the time at half the readout. From a first-order Taylor expansion of the isochromat position at the vicinity of *t* = 0, the previous equation becomes at echo time TE:

$$\begin{array}{c} \phi \left(r,TE\right)=\phi_{0}+\gamma r_{0}\cdot \underset{M_{0}}{\underset{︸}{\int_{0}^{TE}G\left(t\right)dt}}+\gamma u_{0}\cdot \underset{M_{1}}{\underset{︸}{\int_{0}^{TE}tG\left(t\right)dt}}, \end{array}$$ (7)

where *ϕ*_0_ is an additional background phase induced by the initial phase and field inhomogeneities, **u**_0_ the isochromat velocity, and *M*_0_, *M*_1_ the zeroth- and first-order moments of the gradient. Amongst the several existing velocity encoding strategies [32], the so-called flow compensation technique is often preferred as it reduces pulsatile flow artifacts [4]. It consists in a reference scan where all the velocity-induced phase shifts are refocused at the echo time (*M*_0_ = 0, *M*_1_ = 0). Then a second scan is applied with added bipolar gradient to encode the flow velocity (*M*_0_ = 0, *M*_1_ ≠ 0) while removing the background phase shift. This encoding strategy is illustrated in Fig 3. The velocity component along each encoding direction *i* can then be retrieved from the phase difference between these two scans, such as:

$$\begin{array}{c} u_{i}=\frac{V_{enc,i}}{\pi }\Delta \phi_{i}, \end{array}$$ (8)

where Δ*ϕ*_*i*_ = arg(*I*_*i*_) − arg(*I*_*ref*_) refers to the motion-induced voxel dephasing, *I*_*ref*_ corresponds to the reference scan phase image, and *I*_*i*_ to the *i*-th velocity component phase image. $V_{enc,i}=\frac{\pi }{\gamma \Delta M_{1}}$ corresponds to a 2*π* phase shift and is a user-defined parameter. In order to avoid phase wrapping, VENC should be set larger than the largest velocity value expected in the imaging domain, in the given direction and in absolute value. In phase-contrast MRI, acceleration and higher-order terms are neglected due to the first-order Taylor expansion of the isochromat position [33]. Since cardiovascular flows are pulsatile, the cardiac cycle can be sampled into several time-frames (or phases). As the time duration of one cardiac cycle is not sufficient to acquire all the data, the k-space is filled progressively over several cycles, each phase data being acquired in a synchronized way from one cycle to another. This synchronisation is usually performed by using electrocardiogram signal (ECG-gating).

### 2.6 Image reconstruction

A particular property of the MR signal (see Eq 3) is that it can be written as the Fourier transform of the weighted transverse magnetization, using the k-space formalism described in Sec. 2.4. Therefore, the k-space signal can be converted into a 2D or 3D image in the spatial domain by applying a 2D or 3D inverse Fourier transform. More details about signal detection and image reconstruction concepts can be found in [34].

### 2.7 RF-spoiling

The RF-spoiling corresponds to the disruption of the residual transverse magnetization that remains at the end of each repetition, before the next RF pulse is applied. This is generally required in PC-MRI sequences to ensure that the transverse magnetization recovers a steady state before each RF excitation. However, the relaxation of the longitudinal magnetization is generally incomplete. After several repetitions the longitudinal magnetization is saturated and converges towards its steady-state value $M_{z}^{ss}$ generally smaller than *M*_0_ [34]. In practice, RF-spoiling is performed by varying the phase of the RF-pulse following a predefined pattern [35].

## 3 Numerical methods

### 3.1 4D Flow MRI simulation procedure

**Fig 4:**
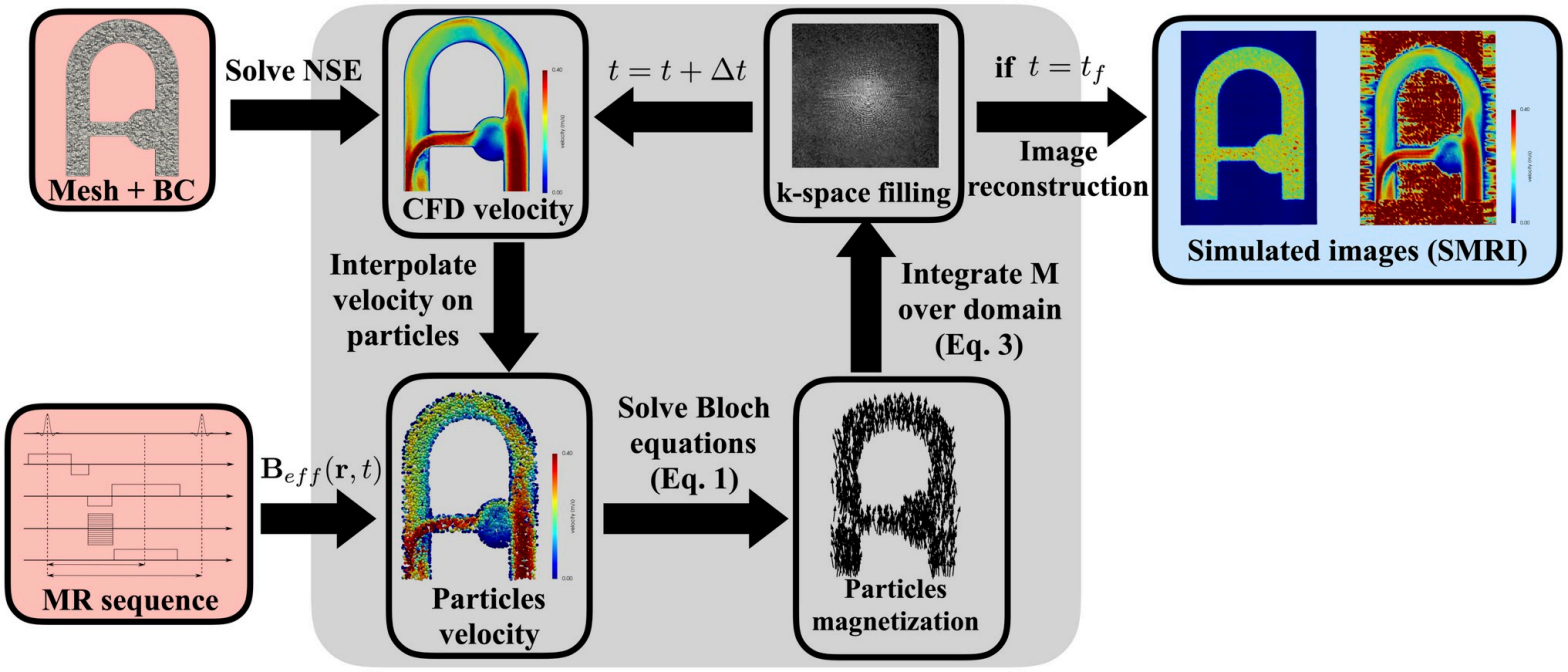
Main steps of the CFD-MRI simulation procedure. NSE: Navier-Stokes Equations. BC: Boundary conditions. The grey block corresponds to the simulation framework kernel, while the red/blue blocks are inputs/outputs to the simulation. *t*_*f*_ is the final time of the simulation. The output “simulated images” correspond to three phase difference images and a magnitude image.

The entire CFD-MRI simulation procedure is illustrated in Fig 4. A pseudo-code of the procedure is provided in Algorithm 1. In our algorithm, the fluid flow is solved independently of the Bloch equations. The Bloch equations are solved on fluid particles that behave as tracers, perfectly following the local fluid velocity. Each particle represents an isochromat, with a value of magnetization advanced in time. All calculations regarding the MRI simulation are performed only on particles. The next sections detail the different steps presented synthetically in Fig 4 and Algorithm 1.

**Algorithm 1:** Pseudo-code of the coupled CFD-MRI simulation procedure.

**Input**:

*B*_1_(*t*), **G**(*t*), *t*, *readout* ← InputSequence()

*T*_1_, *T*_2_, *γ*, **M**_*inj*_ ← ParticlesData()

**r**_*nodes*_ ← MeshData()

**Initialize Data**:

**r**^*p*^ ← FillWithParticles()

**for** *p* = 1: *n*_*pt*_ **do**

 **M**^*p*^ = **M**_*inj*_

 **u**^*p*^ = **u**_0_

**end**

**Temporal Loop**:

**while** *not done* **do**

 *t*^*m*^ = *t*^*n*^

 Δ*t*_*cfd*_ ← CalcCFLTimeStep(*CFL*)

 *t*^*n*^ = *t*^*n*^ + Δ*t*_*cfd*_

 **for** *i* = 1: *n*_*nodes*_ **do**

  **U**^*i*^ ← SolveNavierStokes(**U**^*i*^, Δ*t*_*cfd*_)

 **end**

 **if** *spoiling*==*True* **then**

  DeactivateAllParticles(*n*_*pt*_)

  *n*_*pt*_ ← FillWithParticles()

 **end**

 **while** *t*^*m*^ < *t*^*n*^ + Δ*t*_*cfd*_ **do**

  **for** *p* = 1: *n*_*pt*_ **do**

$B_{eff}^{p}\leftarrow$ CalcBeff($B_{1}^{m},G(t^{m})$)

   $\Delta t_{mri}^{p}\leftarrow$ CalcMRTimeStep($B_{eff}^{p}$)

   $t^{m}=t^{m}+\Delta t_{mri}^{p}$

   **u**^*p*^← InterpolateVelocityOnParticle(**U**)

   **r**^*p*^←AdvanceParticlePosition(**r**^*p*^)

   **M**^*p*^←SolveBloch($B_{eff}^{p},\Delta t_{mri}^{p}$)

 **end**

  **if** *readout*==*True* **then**

   *S*(*t*^*n*^)← CalcMRSignal(**M**^*p*^)

  **end**

 **end**

**end**

#### 3.1.1 Particle seeding

To model the isochromats, a homogeneous spatial distribution of *N*_*p*_ Lagrangian particles is seeded inside a fluid domain which is itself discretized with a fixed (Eulerian) numerical mesh. *N*_*p*,*el*_ particles are seeded inside each Eulerian cell of the mesh following a uniformly random distribution [36]. As found in [37], this randomization should prevent spurious rephasing artifacts to appear. An injection magnetization **M**(*t* = 0) = **M**_**inj**_ = (0, 0, *M*_*inj*_) is prescribed as initial condition for each seeded particle. The longitudinal magnetization is set to its steady state value $M_{inj}=M_{z}^{ss}$ so that no presaturation of the magnetization is required [34]. An isochromat volume $w^{p}=\frac{V_{el}}{N_{p,el}}$ and a set of magnetic properties (*T*_1_, *T*_2_, *M*_0_) are associated to each particle *p* inside an element of volume *V*_*el*_.

#### 3.1.2 Spoiling modeling

As already mentioned in Sec. 2.5, RF-spoiling is generally performed in phase-contrast sequences to remove the transverse residual magnetization. However, as found in [24], a realistic simulation of an RF-spoiling event yielding an error inferior to 3% may require 1000 isochromats/voxel to avoid constructive vector summation and the associated spurious signal. This would lead to a dramatic increase of the computational burden. To circumvent this issue, RF-spoiling is modeled by setting the transverse magnetization of each particle to zero.

Additionally, to take advantage of RF-spoiling, all the particles within the domain are suppressed at each spoiling event, and *N*_*p*_ particles are re-seeded at the same location as initially, with the magnetization vector reset to its initial value $M_{\text{inj}}=(0,0,M_{z}^{ss})$.

**Fig 5:**
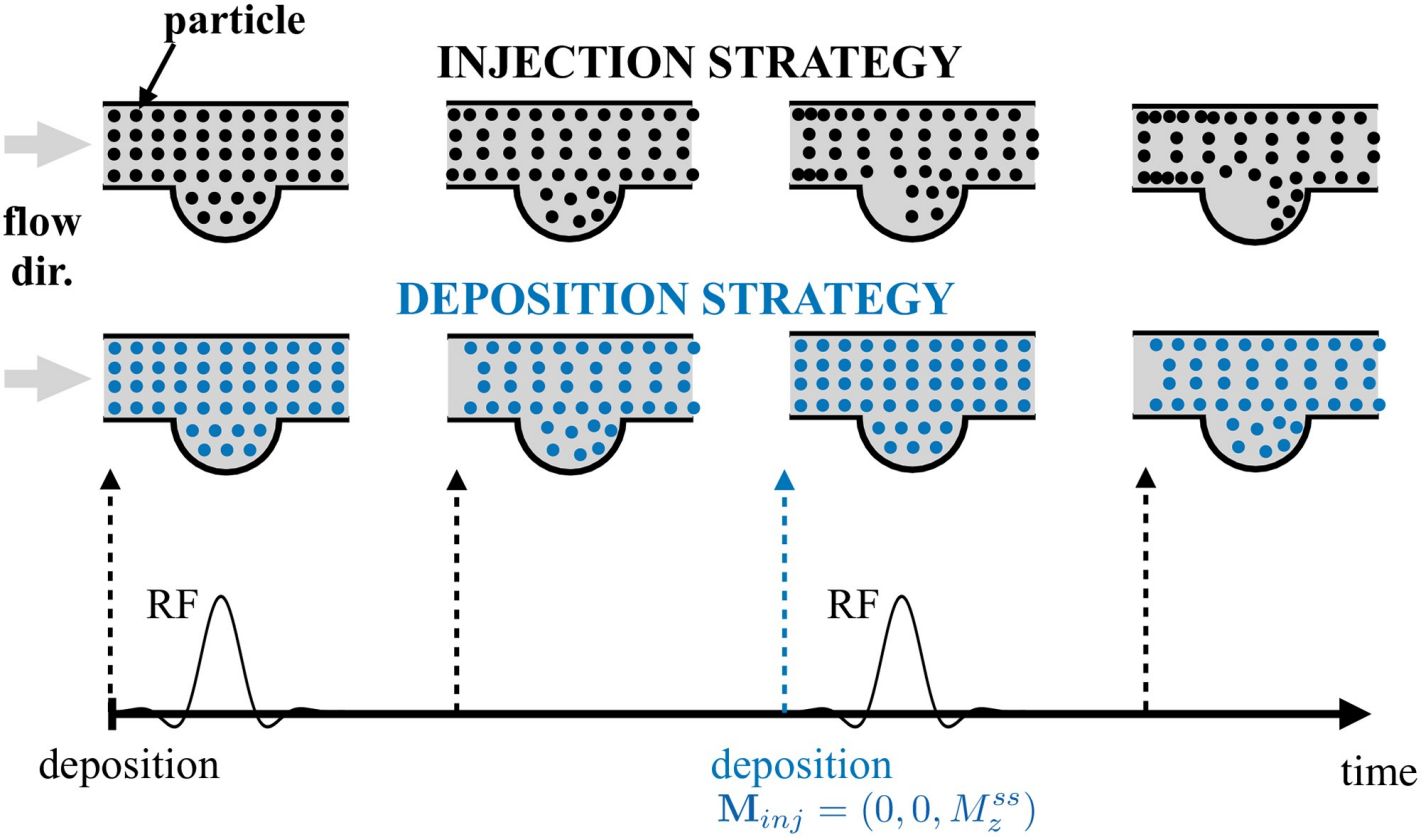
Schematic illustration of the particles position at different instants between two consecutive RF excitations in a pipe flow with an aneurysm-like wall bulging. The black spheres represent the classical particles injection strategy where particles are initially seeded within the whole domain and continuously injected from the inlet boundary surface. In contrast, in the proposed seeding strategy (blue spheres), the particles are periodically re-seeded at their initial location, and the steady-state magnetization is prescribed. In the classical strategy, zones of agglomerated and empty particles can be observed especially in the aneurysm sac [23], while the proposed approach allows to keep homogeneous the particle repartition.

The re-seeding of particles at the same initial location is illustrated in Fig 5. It is a key step of the methodology that presumably allows to keep the particles distribution homogeneous, and avoids areas of spurious signal due to either a lack of particles in high velocity regions, or the accumulation of slow velocity particles near the boundary walls.

#### 3.1.3 Temporal discretization

Owing to the broad variety of magnetic-related time discretization constraints, a multi-criterion time-stepping approach was implemented to numerically solve the Bloch equations. The aim is to ensure the numerical stability and the precision of the algorithm and to properly represent the highest frequencies of the external magnetic field.

During magnetic events when RF is off, a numerical stability criterion for the explicit Runge-Kutta scheme can be obtained from a stability analysis [38], which enforces the following time step constraint:

$$\begin{array}{c} \Delta t_{mri}\leq \Delta t_{stab}=\frac{2}{T_{2}(\frac{1}{T_{2}^{2}}+{\left(\frac{\gamma }{2\pi }B_{z,max}\right)}^{2})}, \end{array}$$ (9)

where *B*_*z*,*max*_ corresponds to the maximum z-component of the magnetic field prescribed to a particle. In addition, to capture the stiff variations of the magnetization induced by abrupt changes in the magnetic field source term, another time step constraint is calculated as:

$$\begin{array}{c} \Delta t_{mri}\leq \Delta t_{mag}=\frac{2\pi b_{n}}{\gamma B_{eff,max}}, \end{array}$$ (10)

where *B*_*eff*,*max*_ corresponds to the maximum effective magnetic field imposed to a particle, and *b*_*n*_ (for Bloch Number) is a dimensionless coefficient fixed by the user which corresponds to the fraction of revolution described by the spin with maximum precessing frequency during one iteration.

When the particle experiences a rising gradient field, the following additional time constraint applies:

$$\begin{array}{c} \Delta t_{mri}\leq \Delta t_{grad}=\underset{i}{\text{min}}(0.1\frac{G_{i,max}}{\left|\frac{\partial G_{i}}{\partial t}\right|}), \end{array}$$ (11)

where *G*_*i*,*max*_ is the maximum gradient amplitude specified in the sequence along the *i*-th axis. This constraint ensures that each gradient ramp is sampled with at least ten time steps. It was notably added for instants with small or null **B**_**eff**_ and with large time derivative of the gradient.

Another time constraint Δ*t*_*seq*_ was imposed to ensure a sufficient sampling of the RF waveform, as well as the correct time delays between readout samples, where the signal is collected.

Finally, a regular update of the information between Eulerian and Lagrangian data was imposed to ensure the coupling with CFD. In practice, the time step was adjusted to match each CFD time step Δ*t*_*CFD*_.

**Fig 6:**
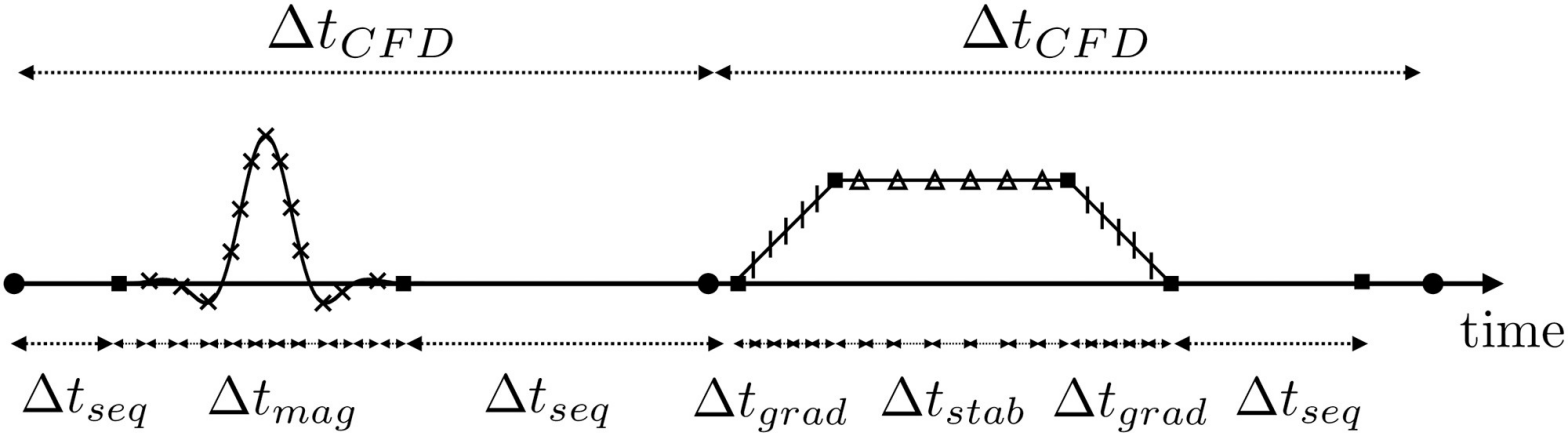
Evolution of the simulation time step over an arbitrary pulse sequence (RF and gradient) as a function of the magnetic event. Within each CFD iteration of time Δ*t*_*CFD*_, the fluid velocity is kept constant. For indication, in a pipe of 5 cm radius and 10 cm length, with *T*_2_ = 10 ms, and with a gradient strength *G*_*max*_ = 10 mT/m of 0.1 ms rise time, the order of magnitude for the minimum time steps would be: Δ*t*_*stab*_ ∼ 10^−7^ s, Δ*t*_*mag*_ ∼ 10^−5^ s (with *b*_*n*_ = 1), Δ*t*_*grad*_ ∼ 10^−5^ s, Δ*t*_*seq*_ ∼ 10^−3^ s and Δ*t*_*CFD*_ ∼ 10^−3^ s.

This multi-time step integration strategy significantly reduces the overall computation time as compared to a classical uniform time stepping strategy for all the equations. For example, a simulation with uniform time steps of Δ*t* = 10^−5^ *s* requiring 20 000 iterations and 294 s to complete would run in 11.4 s and 1218 iterations with the proposed variable time-stepping approach. A typical time step distribution is illustrated in Fig 6, where it is shown that different time step constraints will limit the time step to advance the Bloch equations depending on the events in the sequence.

#### 3.1.4 Coupling with CFD

To advance the particle position, the fluid velocity is calculated, then interpolated to the particles. The fluid velocity is predicted by solving the incompressible Navier-Stokes equations (NSE) that read:

$$\begin{array}{c} \nabla \cdot u=0, \end{array}$$ (12)

$$\begin{array}{c} \rho (\frac{\partial u}{\partial t}+u\cdot \nabla u)=-\nabla p+\mu \nabla^{2}u, \end{array}$$ (13)

where **u**, *p*, *ρ* and *μ* are the velocity field, pressure field, constant density and constant dynamic viscosity of the fluid, respectively. To this aim, the NSE are discretized on a fixed numerical mesh and solved using the YALES2BIO solver [39] (http://imag.umontpellier.fr/~yales2bio/), an in-house CFD solver dedicated to the simulation of blood flows in complex geometries at both macroscopic and microscopic scales [40–44]. The flow solver uses high-order finite-volume non-dissipative numerical methods to solve the NSE on unstructured meshes [45]. All these features are implemented with the coupling of OpenMP and MPI interfaces to confer its massively parallel capabilities to the solver. A fourth-order explicit Runge-Kutta time advancement scheme is used to advance the fluid velocity, as well as a centered fourth-order scheme for the spatial discretization. The divergence-free condition is met thanks to a fractional-step algorithm [46], and the associated Poisson equation is solved using a Deflated Preconditioned Conjugate Gradient algorithm [47]. For turbulent and transitional flows, Large Eddy Simulation are performed, using the sigma eddy-viscosity-based subgrid-scale model [48] to account for the effects of the unresolved sub-grid scales on the dynamics of the resolved structures. More details can be found in [30]. For numerical stability purposes, the CFD time step Δ*t*_*CFD*_ is systematically computed to ensure that the Courant–Friedrichs–Lewy (CFL) condition remains inferior to 0.9. The CFD velocity is then interpolated on each particle with an inverse distance weighting interpolation, and the particle position is advanced as:

$$\begin{array}{c} r^{p}\left(t+\Delta t_{mri}\right)=r^{p}\left(t\right)+\int_{t}^{t+\Delta t_{mri}}u\left(r^{p},t\right)dt, \end{array}$$ (14)

where Δ*t*_*mri*_ is the time step computed as detailed in Sec. 3.1.3, **r**^*p*^ the particle position and **u** the interpolated velocity. The integration is performed using a third-order Runge-Kutta method (RK3). Note that the numerical time step Δ*t*_*mri*_ is associated to the particle advancement which depends on the discretization of the Bloch equations, and differs from the CFD-related time step Δ*t*_*CFD*_, as discussed in the former section. The magnetic time constraints are generally more restrictive than the fluid time steps, by up to three or four orders of magnitude in some cases. In order to avoid redundant CFD calculations, the fluid velocity is kept constant until the sum of the magnetic time steps reaches Δ*t*_*CFD*_.

#### 3.1.5 Numerical advancement of the Bloch equations

So far, two classes of approaches have been adopted in the literature to solve the Bloch equations. The first approach was initially developed in the work of Bittoun et al. [14] for static tissues simulations and later extended to the flow-related effects [15, 21]. In this method, the driving magnetic field is decomposed as a series of piecewise constant waveforms (rectangular RF pulses and gradients). While it requires a relatively low computational effort due to the analytical formulation, a very large sampling frequency is necessary to limit the approximation errors when simulating realistic non-rectangular RF-pulse and gradient shapes. Another classical approach is based on a complete numerical integration, where an iterative method is used to approximate the Bloch equations, with no preliminary assumption on the magnetic field waveform [17, 19, 24]. This resolution method is relatively simple to implement and its accuracy depends upon the order of the time stepping method and of the time resolution. The numerical integration results in higher computational cost as compared with an analytical formulation and it can be unnecessarily time consuming in regimes where the Bloch equations admit exact solutions, particularly during relaxation.

In this study, a semi-analytic solution was implemented. The Bloch equations are solved numerically by using a fourth-order Runge-Kutta scheme (RK4) during the RF excitations, and analytically whenever the particles experience a relaxation or encoding gradients events. Considering *t* = 0 as the end of the RF pulse (where *B*_*x*_(*t*) = *B*_*y*_(*t*) = 0 for all instants such that 0 < *t* < *t*^*n*^ where *t*^*n*^ is the time of the current iteration), the Bloch equations for the transverse magnetization can be expressed as:

$$\begin{array}{c} \frac{dM_{xy}}{dt}=-(\frac{1}{T_{2}}+i\gamma B_{z})M_{xy}, \end{array}$$ (15)

where *M*_*xy*_ = *M*_*x*_ + *iM*_*y*_ and *i*^2^ = −1. The previous equation admits the following solution:

$$\begin{array}{c} M_{xy}\left(r^{p},t^{n}\right)=\left|M_{xy}\left(r^{p},0\right)\right|e^{i\phi_{0}}e^{-t^{n}/T_{2}}e^{-i\phi (r^{p},t^{n})}, \end{array}$$ (16)

where, in the ideal case where off-resonance effects are neglected (Δ*B*_*z*_ = 0), it is recalled that:

$$\begin{array}{c} \phi \left(r^{p},t^{n}\right)=\gamma \int_{0}^{t^{n}}r^{p}\left(t\right)\cdot G\left(t\right)dt, \end{array}$$ (17)

and *ϕ*_0_ is the phase of the transverse magnetization at the end of the RF pulse. To reach an explicit form of the phase, it is further assumed that the gradient profile over time can be properly represented by a piecewise linear function over a set of time intervals [*t*^*m*^, *t*^*m*^ + 1]. Under this assumption, *G*(*t*) is explicitly written as:

$$\begin{array}{c} G\left(t\right)=G\left(t^{m}\right)+{\frac{\partial G}{\partial t}|}_{m}\left(t-t^{m}\right), \end{array}$$ (18)

over each [*t*^*m*^, *t*^*m*^ + 1] interval, and the phase expression can be recast as:

$$\begin{array}{c} \phi \left(r^{p},t^{n}\right)=\gamma \sum_{m=0}^{n-1}\int_{t^{m}}^{t^{m+1}}r^{p}\left(t\right)\cdot G\left(t\right)dt. \end{array}$$ (19)

As already mentioned in Sec. 2.5, an inherent limitation of PC-MRI sequences is the first-order expansion of the isochromat position **r**^*p*^ assumed to reconstruct the velocity. In other words, velocity is assumed constant over each time interval (acceleration and higher order terms are neglected [4]) and the following expression is used:

$$\begin{array}{c} r^{p}\left(t\right)=r^{p}\left(t^{m}\right)+u_{rk}^{p}\left(t^{m}\right)\left(t-t^{m}\right), \end{array}$$ (20)

where $u_{rk}^{p}$ is obtained from the RK3 particle position advancement in order to conserve the benefits of the 3rd-order accurate particle position advancement scheme (see Eq 14). Introducing the two previous decompositions in Eq 19 yields the following expression for the phase:

$$\begin{array}{c} \phi \left(r^{p},t^{n}\right)=\sum_{m=0}^{n-1}\gamma (a^{m}\Delta t^{m}+b^{m}{\left(\Delta t^{m}\right)}^{2}+c^{m}{\left(\Delta t^{m}\right)}^{3}), \end{array}$$ (21)

where Δ*t*^*m*^ = *t*^*m*+1^ − *t*^*m*^, and

$$\begin{array}{c} \begin{array}{cc} a^{m} & =r^{p}\left(t^{m}\right)\cdot G\left(t^{m}\right),\\ b^{m} & =\frac{1}{2}r^{p}\left(t^{m}\right)\cdot {\frac{dG}{dt}|}_{m}+u^{p}\left(t^{m}\right)\cdot G^{p}\left(t^{m}\right),\\ c^{m} & =\frac{1}{3}u^{p}\left(t^{m}\right)\cdot {\frac{dG}{dt}|}_{m}. \end{array} \end{array}$$ (22)

This formulation can easily be implemented and is valid both when encoding gradients are on or during relaxation processes (where gradients are zero). Indeed, the time step constraint enunciated in Eq 9 does not apply. Here the gradients waveforms are assumed linear for the sake of simplicity but the same reasoning could be adopted for non linear gradients waveforms as long as they can be integrated (like for radial or spiral k-space trajectories). The phase *ϕ* can then be reintroduced in Eq 16 so that the magnetization can be explicitly calculated.

At the end of each iteration, the particle position is updated from the velocity vector estimated by CFD, and this procedure is repeated until the end of the pulse sequence. Images are finally reconstructed using an inverse Fourier transform of the full k-space, and each velocity component retrieved from the phase difference images (as detailed in Sec. 2.5).

## 4 Verification and validation

### 4.1 Validation of the Bloch solver

**Fig 7:**
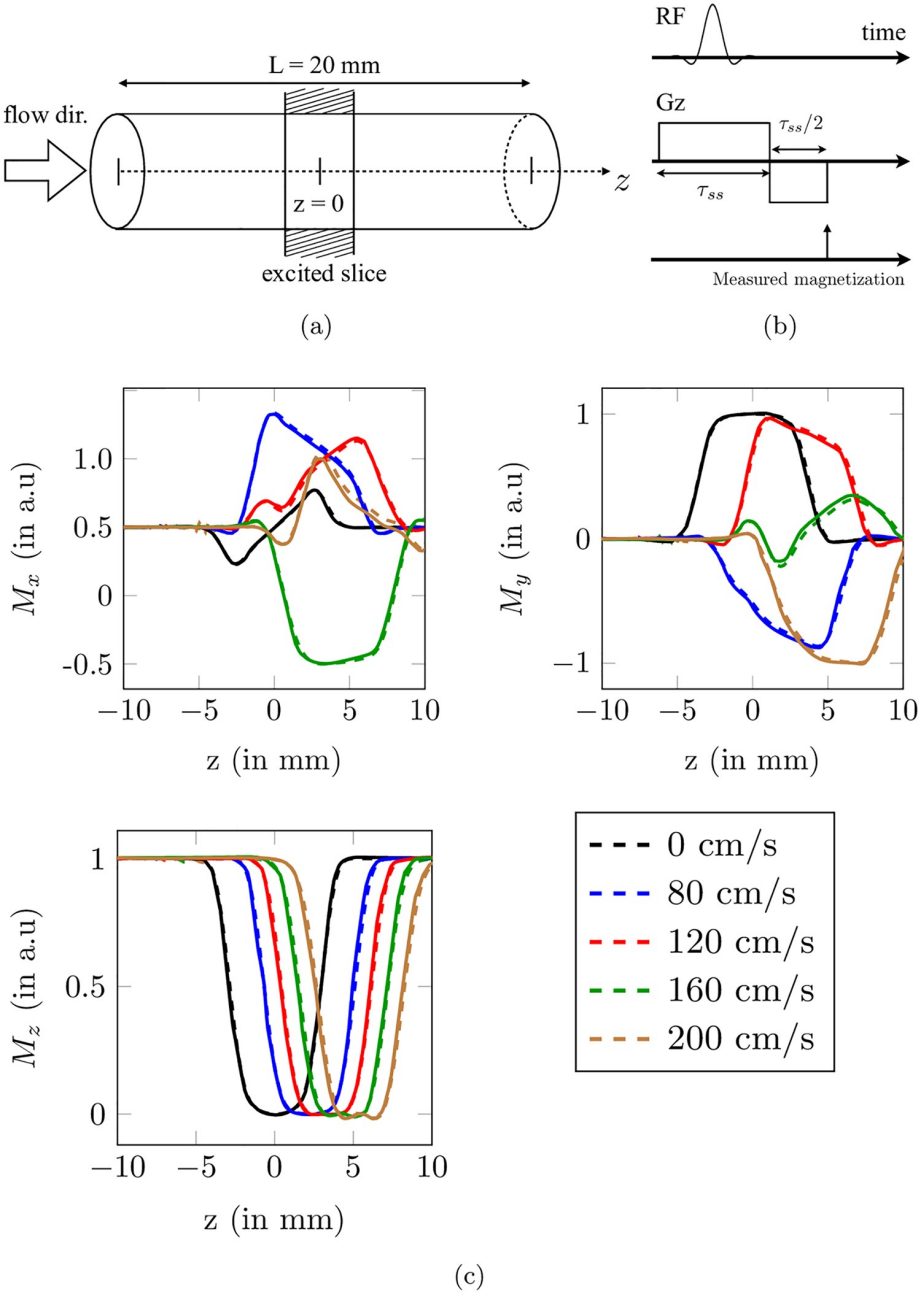
(a) Schematic illustration of the flow configuration used in Yuan et al. [31]. (b) 90° slice selective excitation sequence simulated. (c) Evolution along the centerline of the magnetization (*M*_*x*_, *M*_*y*_, *M*_*z*_) for several velocities imposed to the particles. The dashed lines correspond to the YALES2BIO simulation results, while solid lines correspond to Yuan et al. data [31].

The configuration proposed by Yuan et al. [31] and reproduced in [20] was tested to validate the implementation of the Bloch equations solver. The evolution of the magnetization vector of isochromats flowing along a 1-D segment (see Fig 7a) under a simple 90° slice-selection pulse sequence (see Fig 7(b) was simulated and compared to the results obtained in [31]. It consists in applying an RF-pulse with a limited frequency bandwidth together with a slice selection gradient along the z-axis, which generates a gradient of frequencies around the Larmor frequency and along the *z* direction. In that way, only the spins whose resonance frequency is included in the slice of interest are excited. The magnetization was recorded at the end of the rewinder gradient as marked by the arrow in Fig 7(b). The reported magnetization profiles are compared for several input particle velocities (from 0 to 200 cm/s) in Fig 7(c). The present results are in very good agreement with the outcomes of [31] irrespective of the prescribed velocity. The relative error *ϵ* was computed for different time steps and reached a plateau at Δ*t* = 5×10^−5^ s: *ϵ* ≈ 10^−2^ in the *x* direction and *ϵ* ≈ 10^−4^ for the *y* and *z* components of the magnetization.

### 4.2 Verification of the velocity reconstruction pipeline

**Fig 8:**
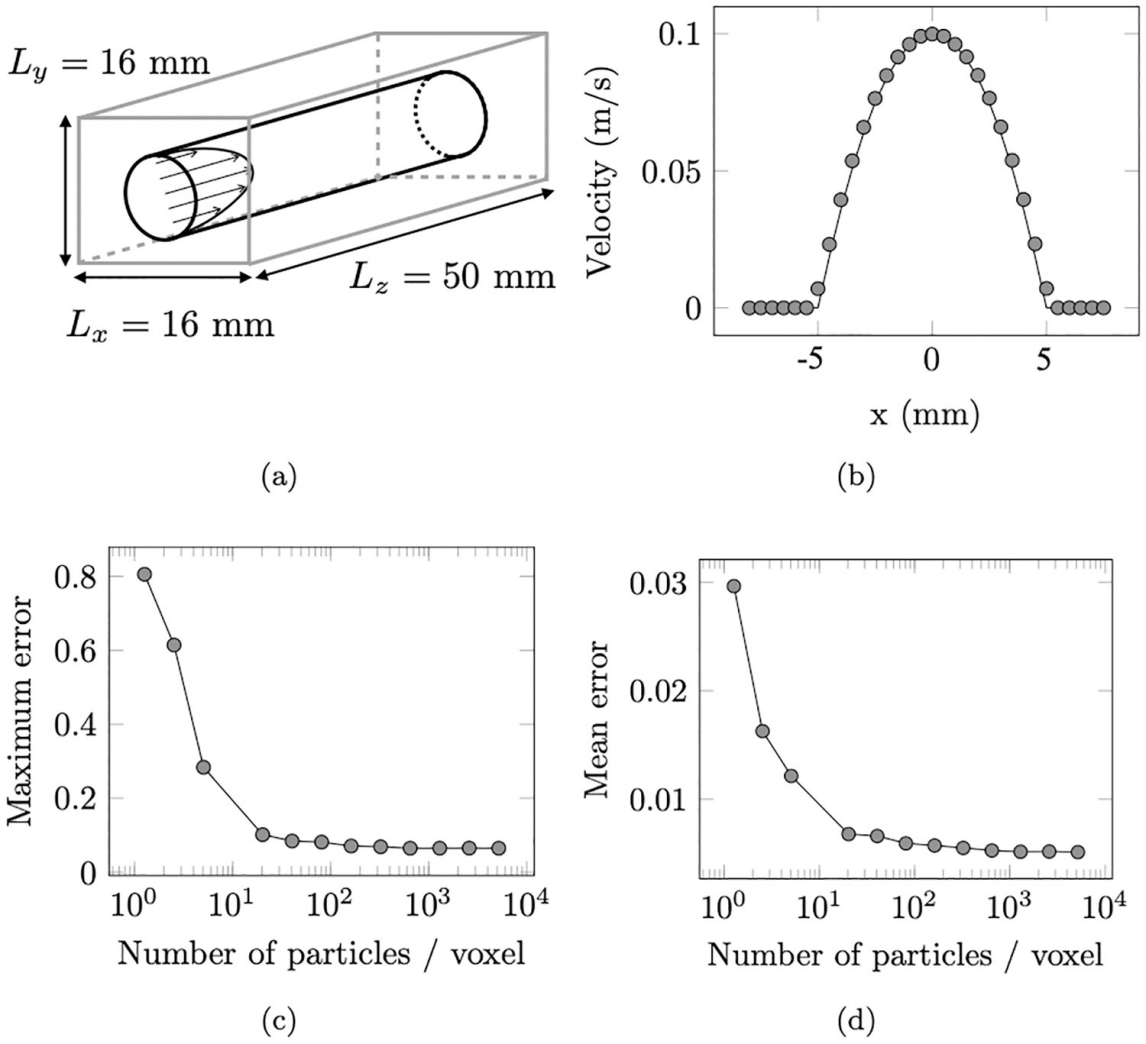
Poiseuille flow test case simulated with a 2D PC-MRI sequence. (a) Boundaries of the domain (flow in black and static tissues in gray) simulated. (b) Axial velocity profile along the *x*-axis reconstructed by MRI (dots) and compared with the imposed Poiseuille analytical solution (solid line). (c) Maximum and (d) mean errors as a function of the particle density.

A second test case was performed to verify the whole velocity reconstruction pipeline. The objective is to retrieve, from an *in silico* 2D PC MRI acquisition, the velocity field prescribed (but not computed) within the region of interest. To this end, a Poiseuille velocity profile was imposed in a duct of square cross-section numerical domain, such that:

$$\begin{array}{c} \begin{array}{cc} w(r) & =w_{max}(1-\frac{r^{2}}{R^{2}})\;\text{if}\;r\leq R,\\ & =0\;\text{elsewhere,} \end{array} \end{array}$$ (23)

where *R* = 5 mm is the radius of the flow domain and *w*_*max*_ = 0.1 m/s the maximum axial velocity. Outside the Poiseuille region (*r* > *R*), a null velocity was prescribed to the spins to simulate static tissues. The numerical domain is illustrated in Fig 8(a). As it gathers both static and moving spins, this configuration allows to mimic the behavior at the interface of a vessel. A 2D PC-MRI sequence was simulated in transverse orientation with a matrix size (*N*_*x*_, *N*_*y*_) = (36, 36), a voxel size Δ*x* = 0.5 mm, Δ*y* = 0.5 mm, Δ*z* = 10 mm, a *FOV* = (18, 18, 18) and a VENC set to 0.12 m/s. Note that a buffer zone was added upstream of the slice position in order to pre-saturate the spins entering the domain, and avoid spurious magnetization inflow effects. Moreover, the field of view was set slightly larger than the numerical domain to avoid wrap-around artifacts [34].

The sensitivity of the velocity reconstruction to the temporal discretization was tested (results not shown, see [38]) by varying the Bloch number *b*_*n*_ (see Eq 10). A large velocity error (*ϵ* = 15%) was found for *b*_*n*_ > 1, followed by a sudden drop for *b*_*n*_ = 1 and a plateau as *b*_*n*_ decreases (*ϵ* = 10^−4^% was found between *b*_*n*_ = 0.25 and *b*_*n*_ = 1). For the present case, the Bloch number was set to *b*_*n*_ = 0.25. To test the convergence of the results with the number of isochromats, up to 4096 isochromats/voxel were seeded in the numerical domain. The image reconstruction was performed by 2D Fourier transform of the collected k-space signal. The velocity field evaluated in the reconstructed image was compared with the analytical Poiseuille flow solution averaged over each voxel.

The axial velocity profile shown in Fig 8(b) reveals an excellent agreement between the analytical and reconstructed velocity fields. A small mean error $\bar{\epsilon }=0.5\%$ was found over the entire image, where the error was defined as: $\epsilon =|\frac{w_{SMRI}-w}{w_{max}}|$, where SMRI corresponds to the MRI simulation. However, slight visual discrepancies can be observed near the boundary walls, where high velocity gradients occur. At this site, a maximum error of max(*ϵ*) = 6.4.% was observed. This can partially be explained by both the finite and discrete sampling of the k-space signal [25]. The former effect is commonly referred to as Gibbs ringing artifact [34] and specifically occurs at regions of sharp phase transition (boundaries of the flow domain).

A convergence analysis with the particle density was undertaken and the results are depicted in Fig 8(c) and 8(d). It shows that about 10 particles/voxel are necessary for the velocity to yield a mean error under 1% as well as about 20 particles/voxel for the maximum velocity error to be lower than 10%. Similarly, the peak velocity at the center of the image raised an error lower than *ϵ* < 1% with only 2 particles/voxel. This result is comparable with the the peak velocity error obtained with a 2D multi-slices sequence and reported in Xanthis et al. [29] (1.6% ± 2.8% error for 24 isochromats/voxel).Generally speaking, these results roughly agree with the study by Shkarin and Spencer [25], where 27 particles/voxel were necessary to keep MR signal error under 1.5% as compared to an analytical signal with stationnary isochromats. However, the reconstructed axial velocity (and not the MR signal) is used here for comparison, thus offering a better validation of the whole pipeline. Besides, the particle density necessary to obtain a consistent signal would be dramatically higher (around 1000 particles/voxel) if the RF-spoiling was simulated instead of modeled, as observed in [24]. Finally, the same computation was performed without the volume weighting fraction associated with the particles. Higher mean errors were produced (5% at 20 particles/voxel), highlighting the importance of accounting for the isochromat volume in the simulations.

#### 4.2.1 Numerical efficiency of the semi-analytic formulation

**Fig 9:**
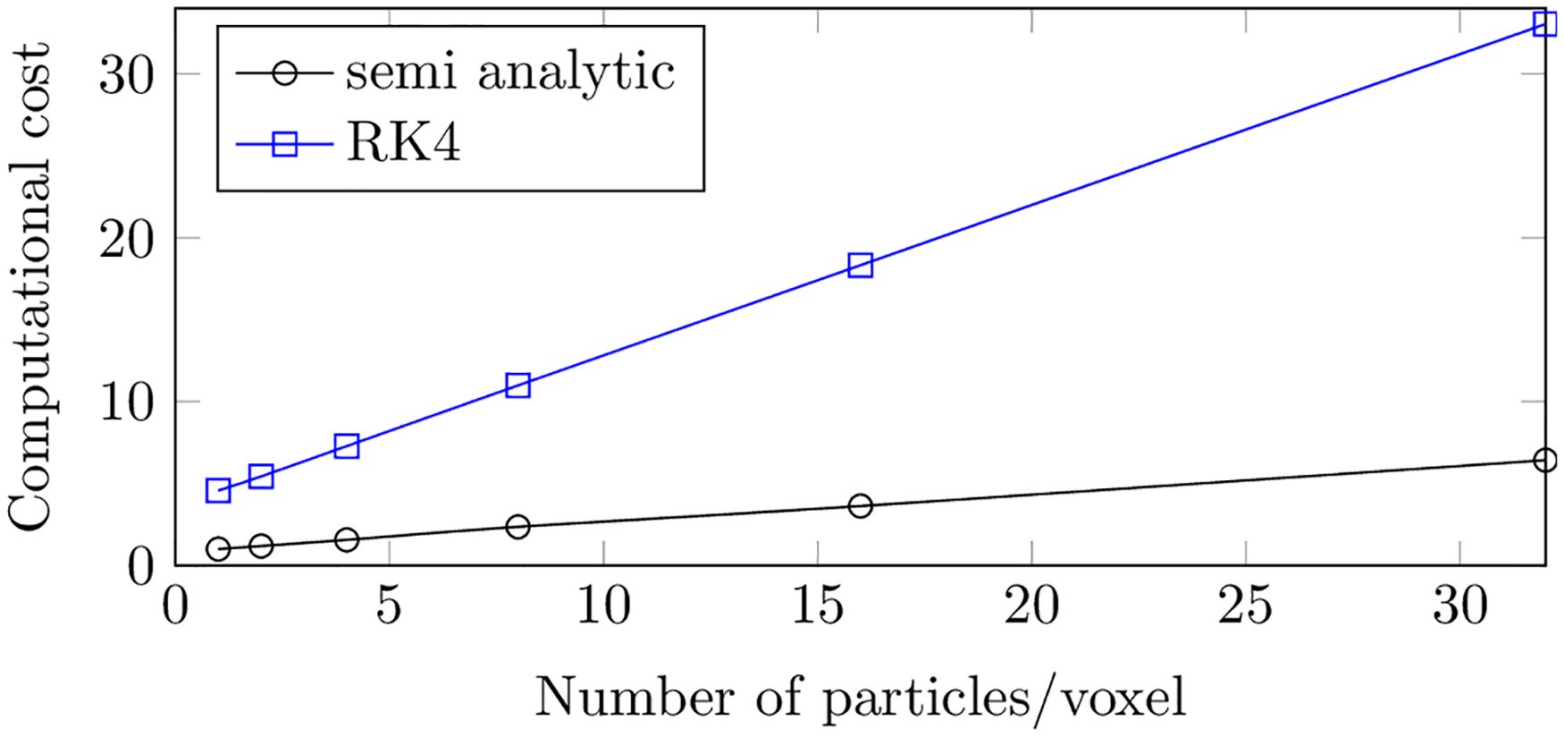
Computational cost for the MRI simulation of a Poiseuille flow with a 2D PC-MRI sequence, at different spin densities with an imaging matrix of size 16 × 16 and a FOV of 32 × 32 × 10 mm^3^. The proposed semi-analytic formulation is compared to the full numerical integration method, where a fourth-order Runge-Kutta numerical scheme is adopted to discretize the Bloch equation. The computational cost is defined as the computational time divided by the lowest time.

To evaluate the computational efficiency of the method, the 2D PC-MRI acquisition of the Poiseuille flow configuration was simulated with the semi-analytic formulation and compared to the velocity reconstructed with the full numerical integration method (RK4). The associated computational costs are presented in Fig 9. While both methods seem to linearly evolve with the number of particles per voxel, the semi-analytic method is 5 times faster while the mean residual errors were the same for both formulations.

## 5 Application to a complex pulsatile 3D flow

The whole CFD-MRI simulation pipeline was tested by simulating the flow phantom experiment presented in a previous publication [30] and briefly detailed thereafter.

### 5.1 Material and methods

#### 5.1.1 Experimental setup

**Fig 10:**
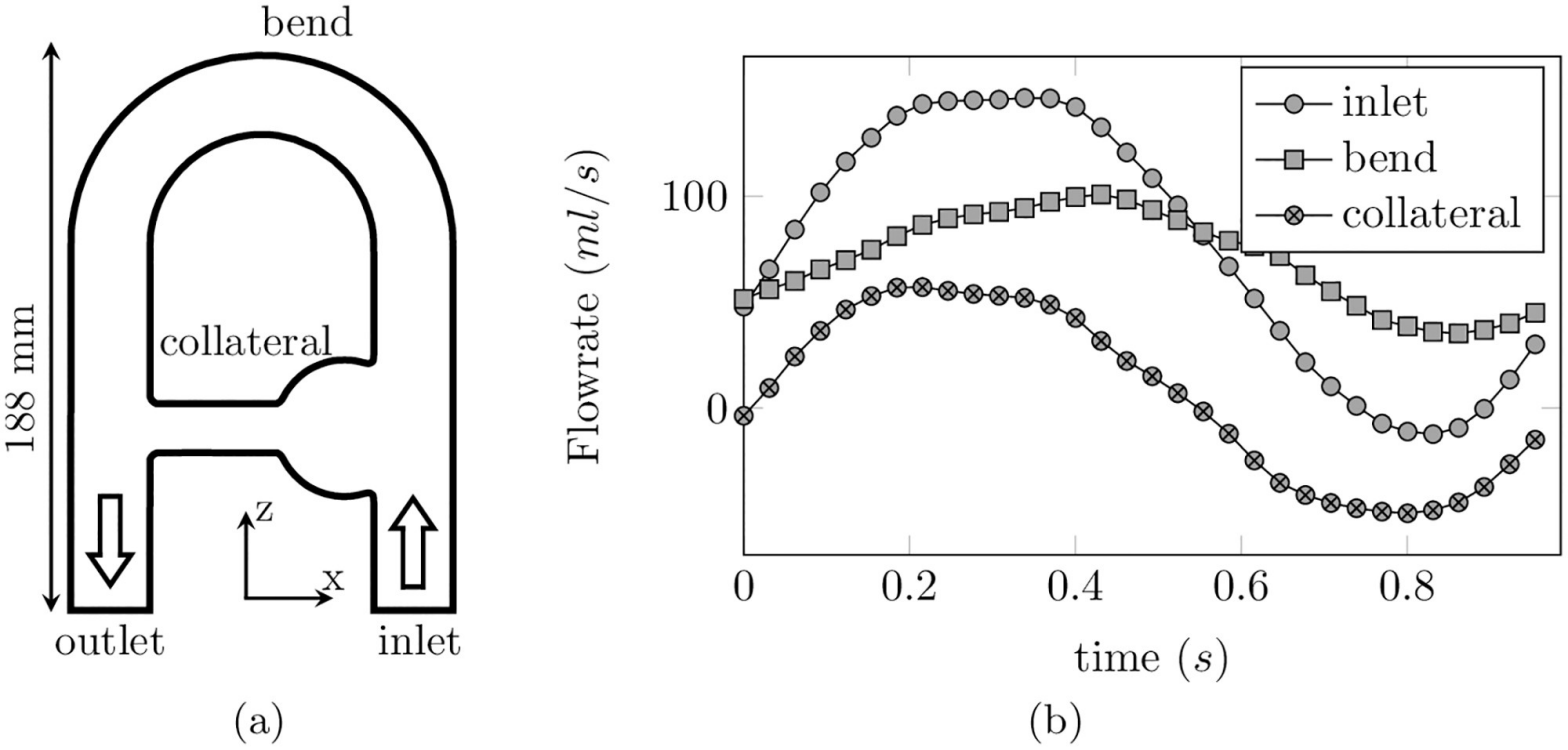
(a) Flow phantom schematic representation annotated with locations of surfaces of interest. (b) Flow rate waveform over a cycle at different locations of the phantom (inlet, bend, collateral).

A rigid flow phantom was constructed to produce complex and realistic flow patterns as observed in the cardiovascular system (see Fig 10a). A separation of flow was introduced to mimic a collateral artery as well as a 180° pipe bend to mimic aortic arch blood flows. A protuberance was also attached at the intersection between the collateral and main branch in order to reproduce the swirling patterns observed in an aortic aneurysm. A pulsatile flow was delivered to the flow phantom by a programmable pump (CardioFlow 5000 MR, Shelley Medical Imaging Technologies, London, Ontario, Canada). A Newtonian blood-mimicking fluid with kinematic viscosity *ν* = 4.02 × 10^−6^ m^2^/s, density *ρ* = 1020 kg/m^3^, and MR relaxation times *T*_1_ = 0.85 s and *T*_2_ = 0.17 s at 1.5 T was supplied to the circuit. More details can be found in [30].

#### 5.1.2 PC-MRI acquisitions

Several scans were carried out with a 1.5 T scanner (Siemens Magnetom Avanto, Siemens Medical Systems, Erlangen, Germany). One 4D Flow MRI scan was performed with prospective gating, full k-space sampling and no parallel imaging acceleration. The encoding velocity was set to *VENC* = 0.5 m/s in all three encoding directions, while *TE* = 3.52 ms and *TR* = 6.6 ms. The matrix size was set to 160 × 160 × 28 and the FOV was 320 × 320 × 56 mm^3^. The spatial resolution was Δ*x* × Δ*y* × Δ*z* = 2 × 2 × 2 mm^3^ while the temporal resolution was Δ*t*_*p*_ = 52.8 ms. Finally, the flip angle *α* was set to 15° and the pixel bandwidth to Δ*f* = 0.6 kHz/Px. This set of parameters resulted in a 40-min scan duration. As detailed in [30], this set of parameters corresponds to an idealized scan as compared to clinical practice setups. It can thus serve as a reference for comparison with the CFD.

An additional 2D cine PC-MRI scan was performed at the inlet surface of the flow phantom, with a high planar spatial resolution (0.78 × 0.78 mm^2^) and a 6mm slice thickness to increase the Signal to Noise ratio. This high-resolution acquisition was used as inflow boundary condition for the CFD computations.

#### 5.1.3 CFD simulation setup

The walls were assumed rigid and a zero pressure boundary condition was prescribed at the outlet. The inlet velocity profile was prescribed from the 2D cine PC-MRI scan acquired at the inlet surface, where the MR velocities were bilinearly interpolated on the numerical mesh at each cardiac phase. A trigonometric interpolation was then performed to fit the time-course velocity signal [30]. The flow phantom was discretized with a tetrahedron-based unstructured mesh with 2 mm characteristic mesh size (approx. 150000 uniform elements), generated with GAMBIT 2.4.6 (ANSYS, Inc., Canonsburg, PA).

#### 5.1.4 Numerical pulse sequence design

A synthetic 4D Flow MRI sequence was designed using the JEMRIS software (http://www.jemris.org/) [17] with characteristics matching the experimental sequence described in Sec. 5.1.2. The acquisition matrix was reduced to (80, 30, 120) as compared to the experimental one to decrease simulation time. Each cardiac cycle of duration *T*_*c*_ = 0.985 s was split into *N*_*p*_ = 17 phases, with a temporal resolution Δ*t*_*p*_ = 58 ms and a repetition time *TR* = 6ms. As the sequence is composed of a reference and three velocity sensitive sub-sequences to fully encode the 3D velocity field [32], it was split into four sub-sequences, each treated separately to reduce the wall-clock simulation time. This technique inherently suppresses the misregistration artifacts that arise in classical interleaved velocity encoding strategies as a result of the time delays between different velocity encoding directions [27]. Therefore, $N_{seg}=\left\lfloor \frac{\Delta t_{p}}{TR}\right\rfloor =9$ subsets (k-space lines) were filled instead of $N_{seg}=\left\lfloor \frac{\Delta t_{p}}{4TR}\right\rfloor =2$ normally with interleaved velocity encoding. The resulting physical time to simulate each subsequence was then $T_{acq}=\frac{N_{y}N_{z}N_{p}}{N_{seg}}\Delta t_{p}=394.4$ s. Such a long sequence yields arrays of few millions entries. For this reason, the sequence reading was segmented so that only small arrays composed by 1000 cells or less are manipulated and stored in data buffers. This decomposition has proven to efficiently accelerate the computations as the buffers are small enough to be stored in the cache memory, therefore avoiding the repeated access to the Random Access Memory [45]. Although the numerical sequence was designed in line with the experimental protocol parameters, it is expected that some divergences remain because the details of the MRI sequence are manufacturer-proprietary information.

#### 5.1.5 MRI simulation setup

The semi-analytic formulation presented in Sec. 3.1.5 was used to solve the Bloch equations, with a Bloch number *b*_*n*_ = 1, in agreement with what was found in Sec. 4.2. To minimize the relative error while keeping a reasonable simulation time, 48 particles/voxel were seeded within the fluid domain following the procedure detailed in Sec. 3.1.1, resulting in about 1.1 million particles seeded in total. All the simulations were performed on Dell PowerEdge C6320 nodes composed of 28 cores Intel Xeon E5-2690 V4 2,6 GHz with 128 GB random-access memory per node. The whole SMRI-CFD simulation was run with twelve nodes in about 20 hours and 33 917 700 iterations.

#### 5.1.6 Data reduction

Several post-processing steps are required to properly compare the CFD velocity field at the nodes of the fixed unstructured grid with the simulated MR velocity images (SMRI). First, as several cardiac cycles are necessary to fully fill the k-space, the CFD velocity field was phase-averaged over the entire simulation time, removing the first ten cycles to cancel the effects of the non-physical initial condition. The phase-averaged velocity field (denoted HR-CFD) was then downsampled to the MRI spatial resolution (denoted LR-CFD). Detailed explanations can be found in [30].

### 5.2 Numerical efficiency of the particle seeding strategy

**Fig 11:**
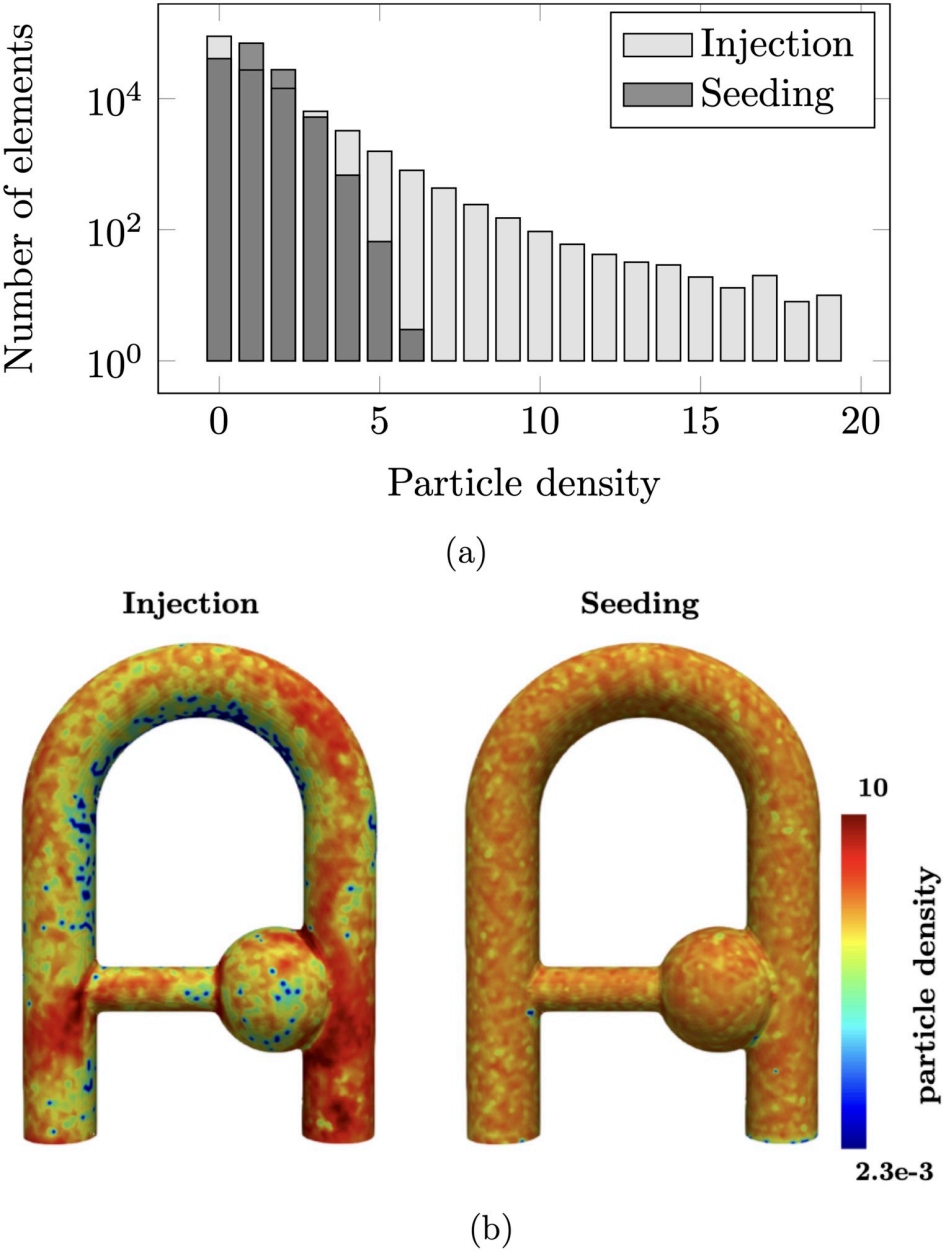
a) Particle density histogram with y axis expressed in logarithmic scale and b) particle density maps over the surface of the flow domain, after a simulation time of 8 s. Note that the particle density was mapped with a log scale to highlight both the dense and sparse regions. The ‘seeding’ strategy is the proposed particle reinitialization strategy while the ‘injection’ approach corresponds to the continuous injection from the inlet boundary surface. Note that only the external surface is shown as the largest zones of particles agglomeration and sparsity are located at the boundary cells.

To highlight its benefits, the proposed particle seeding strategy described in Sec. 3.1.1 was compared with a classical injection approach. In the classical approach, the particles are initially seeded in the whole domain, and continuously injected through the inlet boundary surface [24]. The injection rate is set such that the number of particles is kept constant during the simulation. In the proposed approach, the particles positions are reinitialized after each repetition time. The particle density and sparsity distributions obtained from the two approaches are reported in Fig 11 where the particle density is here defined as the current-to-initial ratio of the number of particle per mesh element. In other words, a homogeneous particle distribution would result in a particle density of 1.0. As compared to the boundary injection approach, the proposed strategy results in a net improvement of the particle distribution homogeneity. The density and sparsity maps shown in Fig 11 reveal significant reduction of the particles agglomeration especially near the entrance and at the collateral elbow, as well as a better particle filling near the inner wall of the bend. Quantitatively, the seeding strategy results in 0.05% of the Eulerian grid cells having a particle density larger than 5 while 4.8% of cells for the injection strategy. Similarly, 28% and 64% of the cells have a particle density lower than 1.0 for the seeding and injection strategies, respectively.

### 5.3 Results

#### 5.3.1 Qualitative analysis

**Fig 12:**
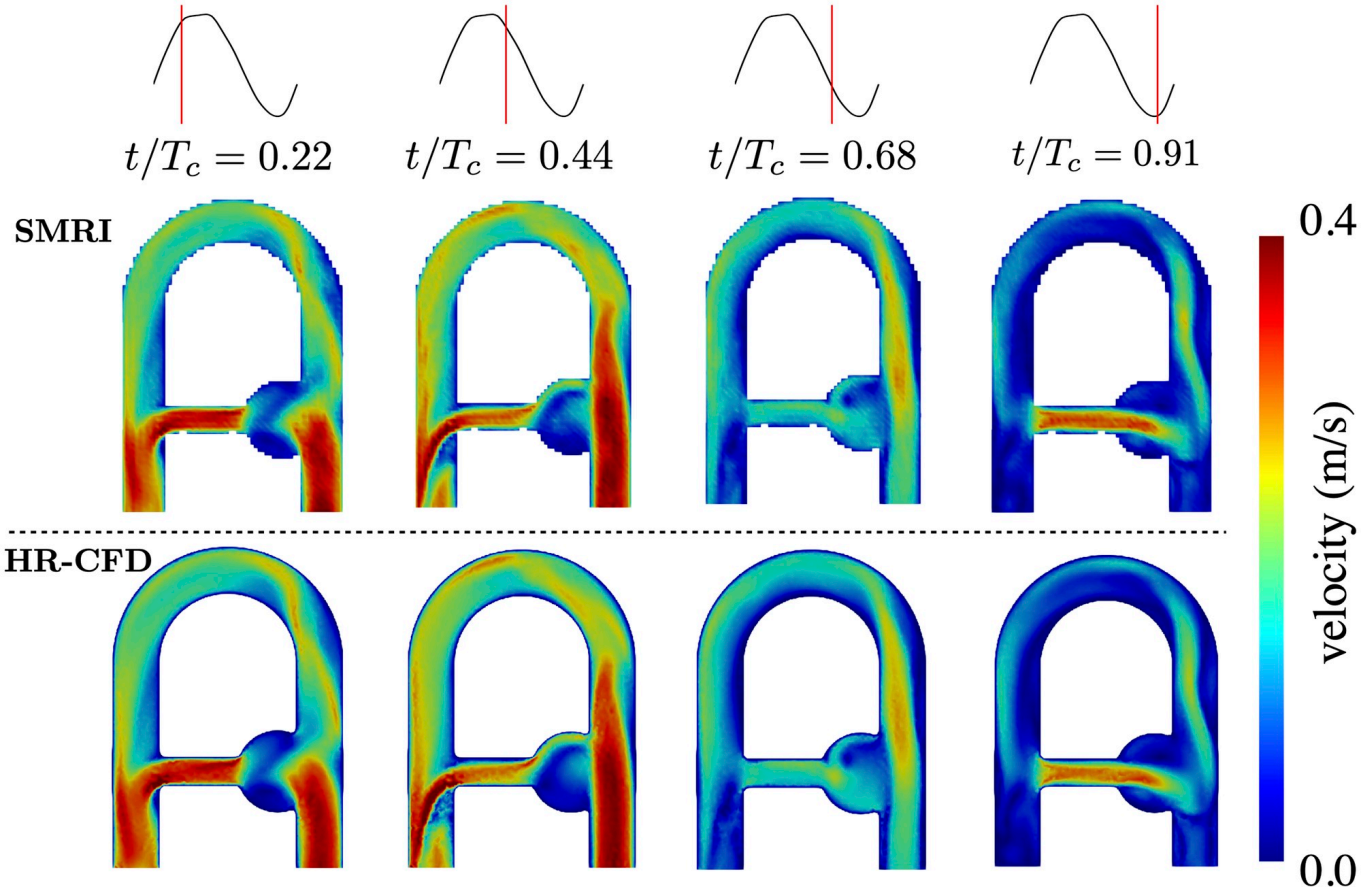
Comparison in the XZ-middle plane between (top) ||u_*SMRI*_|| and (bottom) ||u_*HR*_|| at four different phases during the cycle.

Fig 12 compares the magnitude of the simulated MRI velocity field to the magnitude of the phase-averaged CFD velocity field at different instants in the cycle. For visual clarity, the computed MR images were segmented with a binary mask obtained from the signal magnitude of the simulated MRI. A first visual comparison shows good agreement of the two fields regardless of the phase considered, although the SMRI velocity seems blurred as compared to the CFD field. This may result from the summation of a finite sample of particles. In both cases, the largest spatial velocity variations mainly occur in the pipe bend, aneurysm neck and mixing layer at the collateral outlet elbow. The flow structures smaller than the voxel size should produce intravoxel phase dispersion due to the vector summation of a finite number of spins with different velocity vector directions. It has been shown that three isochromats per spatial directions per voxel are required to reduce the image error under 1.5% [25]; with 48 particles/voxel, the present simulation is expected to keep this source of error to a small value.

#### 5.3.2 Quantitative analysis

**Fig 13:**
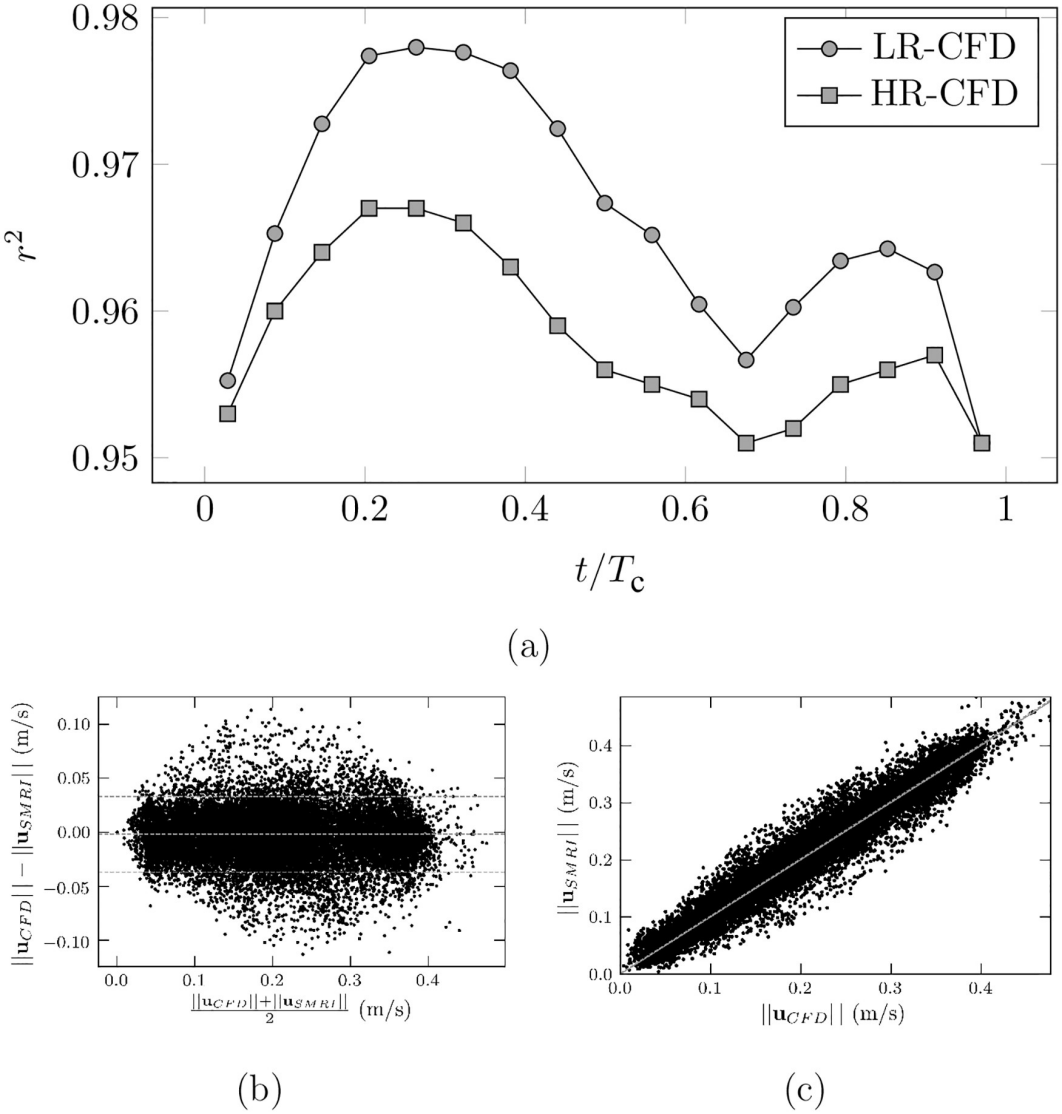
(a). Evolution along a cycle of the Pearson’s correlation calculated between HR-CFD and SMRI, as well as between LR-CFD and SMRI. (b). Bland-Altman and (c). linear regression plots at phase *t*/*T*_*c*_ = 0.2 for the SMRI/LR-CFD comparison.

The Pearson’s correlation coefficient was used as an indicator of the similarities between SMRI and CFD velocity fields. It is defined as the covariance between the two modalities, normalized by the product of their standard deviation. Fig 13 shows the time evolution of the Pearson’s correlation coefficient computed between the SMRI velocity and the CFD phase-averaged velocity field. As expected, the low-resolution CFD velocity better matches the SMRI velocity (peak correlation *r*^2^ = 0.978 and mean correlation *r*^2^ = 0.966) as compared to the high-resolution CFD (peak correlation *r*^2^ = 0.967 and mean correlation *r*^2^ = 0.958). In addition, the highest correlation is reached when the inlet flow rate is maximum. Note that the temporal evolution of the correlation over a cycle roughly follows the same trends as the absolute flow rate (see Fig 10b). Moreover, Bland-Altman and linear regression plots shown in Fig 13 reveal that some discrepancies still remain, with no systematic bias. The effect of the parameters of the SMRI on the errors is now investigated.

#### 5.3.3 Influence of the spin density

**Fig 14:**
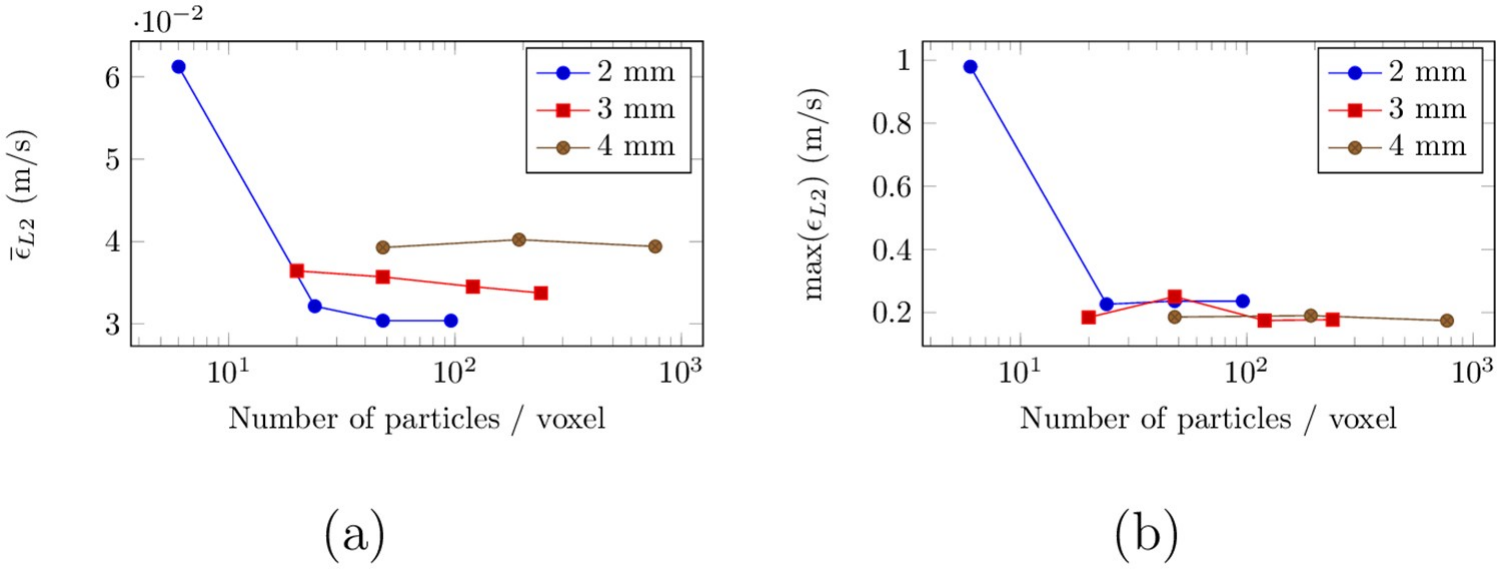
Evolution of the (a) mean and (b) maximum mismatch between LR-CFD and MRI simulation for different spin densities and voxel sizes at *t*/*T*_*c*_ = 0.44.

As for the Poiseuille flow validation case (see Sec. 4.2), the influence of the particle density was assessed. The L2-norm of the mismatch *ϵ*_*L*2_(**r**_*i*_, *t*^*n*^) = ||**u**_*SMRI*_(**r**_*i*_, *t*^*n*^) − **u**_*LR*_(**r**_*i*_, *t*^*n*^)|| was computed as it accounts for the errors owed to both the magnitude and orientation of the velocity vector. The volume-averaged and maximum errors are plotted in Fig 14 as a function of the spin density at time *t*/*T*_*c*_ = 0.44. Let us first consider the baseline case, with a voxel size of 2 mm.

As found for the steady Poiseuille flow simulation in Sec. 4.2, a plateau can be observed around 40 particles/voxel, although a higher mean error is reported as compared to the simple Poiseuille flow. This discrepancy corresponds to about 3 cm/s, or 6% of the VENC. This higher error can most probably be explained by larger spatial misregistration artifacts due to the presence of an accelerated three-dimensional flow. For the maximum error, a similar plateau corresponding to 20 cm/s or 40% of the VENC is reported.

#### 5.3.4 Influence of the spatial resolution

Two additional simulations were performed with voxel sizes set to (3 mm)^3^ and (4 mm)^3^. The resulting volume averaged and maximum L2-norm errors are shown in Fig 14. Similarly to the 2 mm case, the mean error associated with the 4 mm voxel size also reaches a plateau between 20 and 40 spins/voxel. For the 3 mm case, the mean error slowly decreases as the particle density increases. A comparison of all three cases reveals a global increase of the mean error with the spatial resolution. Similar convergence behavior is observed for the three cases when considering the maximum error (see Fig 14b), with a plateau comprised between 40-50% of the VENC. Note however that the maximum error is not impacted by the SMRI resolution.

**Fig 15:**
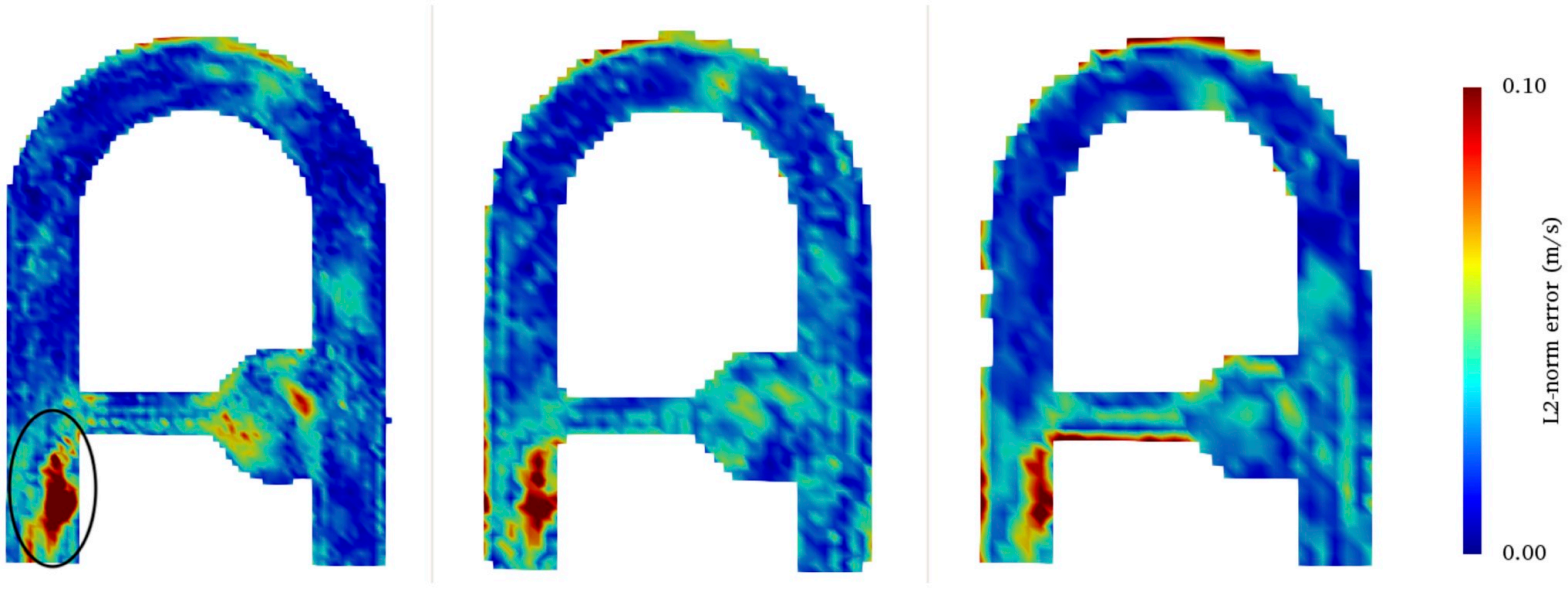
Map of the velocity mismatch at *t*/*T*_*c*_ = 0.44 between LR-CFD and SMRI at three different spatial resolutions. **Left** 2 mm^3^, **middle** 3 mm^3^, and **right** 4 mm^3^. The error is calculated based on the LR-CFD, which is specific to each spatial resolution. In all the simulations, 48 particles/voxel were seeded in the domain.

As errors are calculated with respect to a downsampled CFD, which supposedly reproduces the voxel averaging process, it is not straightforward that larger voxel sizes would induce larger mean error levels. To get an in-depth understanding of this phenomenon, the L2-norm error maps in the XZ middle plane of the phantom are presented in Fig 15. The latter show that, at solid boundaries, high velocity errors are amplified as voxel size increases. This effect mainly arises as a result of the SMRI processing at boundary walls, where the voxel signal is partially averaged by the random phase noise that lies outside the phantom. Consequently, the errors are amplified at larger voxel sizes as a larger proportion of the voxel lays outside the domain. This effect is however not reproduced by the CFD down sampling, where the velocity is averaged with zero velocity contributions instead of random noisy velocity comprised between [−*VENC*, *VENC*]. A simple remedy would be to add stationary particles around the flow phantom to generate a coherent signal, as it was done in the Poiseuille test case (see Sec. 4.2).

Regardless of the voxel size, the other predominant region of large L2-norm errors can be observed downstream of the collateral branch, in the recirculating zone located under the jet (see the circled region in Fig 15). This region harbors highly disturbed flow patterns (especially at this specific phase), where the high momentum of the collateral jet induces a recirculating flow region with adverse pressure gradient and counter rotating vortices. As it will be detailed in Sec. 5.4, the larger errors observed in this particular location are acceleration-induced.

### 5.4 Origin of the measurement errors

An MRI simulator presents the main advantage to produce *in silico* images which are by definition, free of experimental errors. In this sense, one could use the MRI simulation to strictly identify the origins of the measurement errors in 4D flow MRI in order to determine what proportion of the errors is associated with a malfunctioning of the hardware, and which is related to software limitations (reconstruction or pulse sequence). Of course, such a study would necessitate the explicit simulation of real effects and modifications of MRI sequences to isolate errors. This is a study in itself and is out of the scope of the paper. This section should be viewed as an illustration of the capabilities of MRI simulation to point to different types of errors and quantify their effects as a function of the characteristics of the flow of interest.

#### 5.4.1 Software-related errors

**Fig 16:**
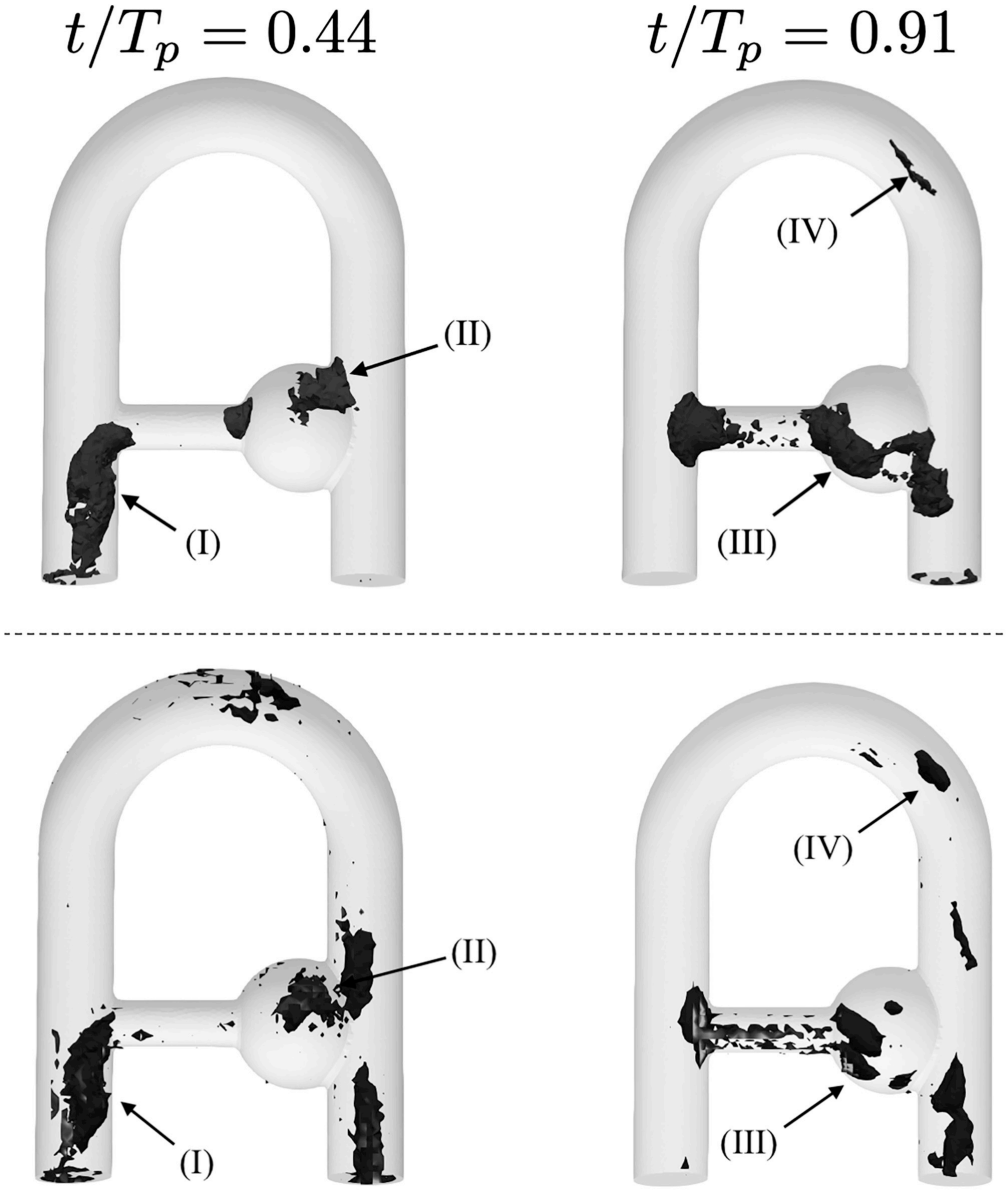
Largest patterns of phase-averaged CFD acceleration (top) and L2-norm of the SMRI/LR-CFD velocity mismatch (bottom) at two instants in the cycle. The threshold values were set to (left) 25 and (right) 7% of the maximum CFD acceleration and (left) 14 and (right) 12% of the maximum mismatch at *t*/*T*_*c*_ = 0.44 − *t*/*T*_*c*_ = 0.91, respectively.

While the *in silico* images are free of noise, with no magnetic field distortion, phantom motion or off-resonance effects, some sources of velocity discrepancies subsist such as intravoxel phase dispersion [49], velocity fluctuations effects [22] or k-space troncature and discrete sampling artifacts. Moreover, as highlighted by Steinman et al. [27], some errors could be caused by spatial misregistrations due to the time delays between the different spatial and velocity encodings, as well as velocity displacement artifacts due to the acceleration of spins during velocity encodings. The former does not exist in the present study since the three velocity components were acquired simultaneously (see Sec. 5.1.4). Conversely, the latter should be prevalent in the highly pulsatile flow regime considered. This is illustrated in Fig 16 which indicates regions of large SMRI/LR-CFD discrepancies and of large phase-averaged acceleration field ($\frac{\partial u}{\partial t}+u\cdot \nabla u$) computed from the HR-CFD field. Similar structures can be observed in the collateral jet (I), in the main flow detachment into the aneurysm neck (II), as well as at the beginning of the pipe bend (IV). These similarities suggest that at least some of the largest velocity mismatches are owed to the non-inclusion of acceleration terms in the phase equation (see Eq 20) by assuming linear time variations of the spin position [50]. Should it be mentioned, this acceleration artefact is inherent to the MRI sequences used in practice and is not related to the methodology proposed to generate *in silico* MRI.

#### 5.4.2 Hardware-related errors

**Fig 17:**
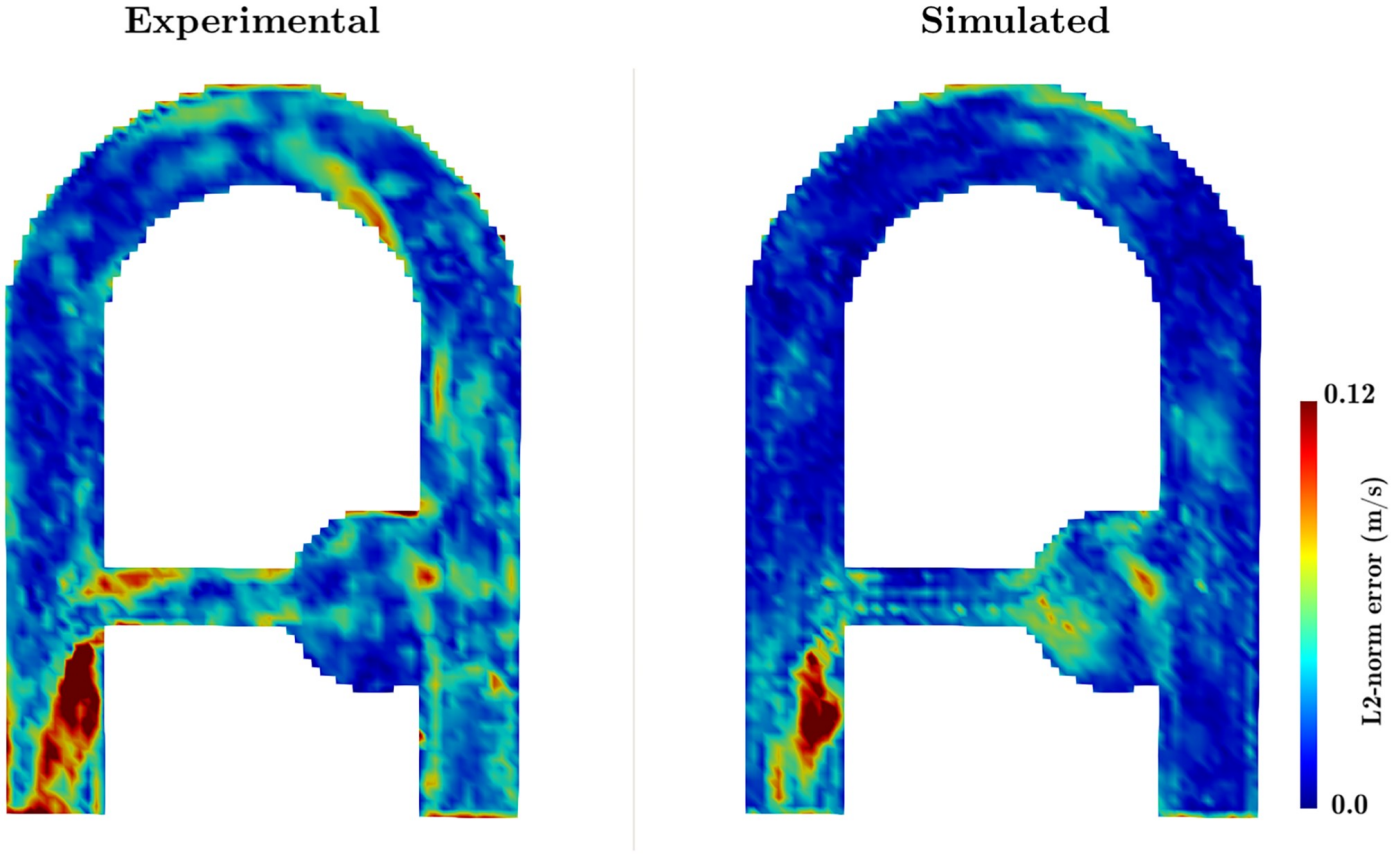
Velocity L2-norm error in the XZ-middle plane of the (left) experimental and (right) simulated MRI at peak systole *t*/*T*_*c*_ = 0.44. The L2-norm error is calculated based on the LR-CFD obtained from a 2 mm characteristic mesh size.

The simulated MRI velocity was also compared with the experimental 4D Flow MRI measurements described in the previous Sec. 5.1.2. The measured and the synthetic velocities were compared to the CFD velocity at peak systole (*t*/*T*_*c*_ = 0.44), when the flow rate is maximum. L2-norm error maps are reported in Fig 17. A first striking result is that the highest error sites are similarly located under the jet, at the collateral elbow outlet. As previously suggested, they mainly arise as a result of the large convective acceleration. Moreover, the MRI error spreads on a larger region as compared to SMRI. It should be recalled that the 3 directions of the velocity were encoded simultaneously in the simulations to avoid time delays between different velocity components. Conversely, the interleaved velocity encoding strategy adopted in the experimental sequence could induce non-negligible time offsets and cause this widespread error pattern in the direction of the main flow. Note that the larger error spots observed in the MRI measurements were expected as the MRI simulations are free of many additional sources of experimental limitations such as magnetic field inhomogeneities, off-resonance effects and measurement noise.

## 6 Discussion

In this study, a workflow for simulating realistic time-resolved 3D PC-MRI acquisitions was presented. To this aim we introduced a numerical procedure with an “on-the-fly” CFD coupling. A semi-analytic solution of the Bloch equations as well as a periodic particle seeding strategy were implemented to accelerate the computations. The computational gain of the semi-analytic formulation was evaluated, as well as the parallel efficiency of the entire program. The Bloch equations solver was validated from the literature [31], and a Poiseuille flow configuration was simulated to validate the full velocity reconstruction workflow. Even for this simple flow configuration with large particle density, a systematic velocity error was reported with peaks around 6% near the boundary walls. It was inferred that the discrete sampling and truncation of the k-space were partially responsible for the remaining error. To cancel these effects, the Poiseuille velocity field might be convoluted with the point spread function described in [34]. To validate the coupling with CFD, several MRI simulations of a well-controlled flow phantom experiment were performed and compared to the phase-averaged CFD velocity downsampled to the MRI resolution. Qualitatively, a very good agreement was observed irrespective of the considered phase. The largest velocity correlation was obtained at peak systole while a decreasing correlation was observed as the flow rate decreases. A potential explanation for this trend is that phases when flow deceleration occurs are very favorable to the development of turbulent-like flow features [43].

The sensitivity of the solution to spatial resolution and spin density was investigated. As compared to the existing simulators, the presented method seems to provide an accurate solution with less particles. We suggest that this is a direct consequence of the proposed particle seeding strategy, which maintains a homogeneous particle distribution along the entire simulation. Besides, the semi-analytic formulation coupled to the massively parallel capabilities of the YALES2BIO solver allows the simulation of realistic flow MRI sequences with physical times up to hundreds of seconds.

As an illustration of the usefulness of *in silico* MRI capabilities, the numerical pipeline was used to discriminate between the measurements errors caused by the pulse sequence limitations and the distortions induced by hardware flaws. It was suggested from the comparison with CFD acceleration maps that larger errors are related to the fact that the spins acceleration is not accounted for in the phase-velocity relationship (see Sec. 3.1.5). Acceleration-sensitive acquisitions could be acquired to account for the flow high-order motion [51] or post-processing corrections could be applied to correct the velocity field [33].

The MRI/CFD mismatch was also compared to the SMRI/CFD mismatch. While the dominant velocity error patterns were well predicted by the simulation, some errors were not reproduced. It implies that some of the observed errors were associated with off-resonance effects such as gradient non-linearities, T_2_* relaxation effects, chemical shift, or magnetic susceptibility.

However, some limitations remain and numerical implementations should be considered to make *in silico* MRI more realistic. First, some numerical artifacts introduced by the modeling assumptions were not addressed and require further developments. For instance, injection of pre-magnetized particles at the inlet boundary surface are necessary to avoid spurious signal resulting from sparsely distributed particles. Moreover, some errors are associated with the cycle-to-cycle fluctuations: for instance the phase-averaged CFD velocity is compared with MR velocity resulting from a progressive k-space filling. Reinitializing the flow field periodically at each cardiac cycle would remove the cycle-to-cycle fluctuations and thus isolate the related errors. This should however produce less realistic MR velocity images. Then, coil sensitivity profiles should appear as a weighting factor of the MR signal to mimic experimental MRI acquisition. The magnetization dynamics can be fully described by solving the Bloch-Torrey equations that accounts for the transfer of magnetization due to diffusion [52]. Also, a relatively coarse CFD numerical mesh was used in the simulations to limit the computational cost. While it should not impact the comparison with the simulated MRI velocity, it is expected that a coarser CFD produces higher velocity deviations from the real flow field, and therefore could yield larger errors as compared to the experimental MR velocity measurements. A finer mesh should be considered to improve the results [30]. Finally, a preponderant limitation of this framework is that there are numerous differences between the simulated and experimentally acquired sequences. For example, the SMRI sequence was designed as a retrospectively gated sequence while the experimental measurements presented were prospectively gated. To this respect, the readout time sampling was slightly different as compared to that of the MRI. Moreover the three velocity components were encoded simultaneously in the simulation, but sequentially in real MRI experiment. As a result, potential time delays between each velocity component could arise and amplify the errors. As it belongs to the manufacturers, little is known about the design of the experimental sequence and further developments are required to simulate realistic MR sequences. Nevertheless, basic pulse sequence blocks can be numerically reproduced with higher fidelity and should suffice to identify the hardware flaws. While the present manuscript is devoted to the description and validation of an MRI simulation framework, future developments will be dedicated to assessing the influence of acquisition parameters such as VENC on the velocity outcomes.
